# Reducing cadmium bioaccumulation in *Theobroma cacao* using biochar: basis for scaling-up to field

**DOI:** 10.1016/j.heliyon.2022.e09790

**Published:** 2022-06-23

**Authors:** Julián E. López, Catalina Arroyave, Adriana Aristizábal, Byrone Almeida, Santiago Builes, Eduardo Chavez

**Affiliations:** a Environmental Engineering Department, Universidad de Medellín, Carrera 87 # 30-65, 050026 Medellín, Colombia; b Process Engineering Department, Universidad EAFIT, Carrera 49 # 7 Sur-50, 050022 Medellín, Colombia; c Universidad Estatal de Milagro, UNEMI, Facultad de Ingenierías, Ciudadela Universitaria km 1 ½, Milagro, Ecuador; d Escuela Superior Politécnica del Litoral, ESPOL, Facultad de Ciencias de la Vida, Campus Gustavo Galindo Km. 30.5 Vía Perimetral, P.O. Box 09-01-5863, Guayaquil, Ecuador

**Keywords:** Soil pH, Cocoa beans, Potentially toxic element, Soil remediation, Bioavailable Cd

## Abstract

The intake of Cd-enriched food is the main Cd pathway for the nonsmoking population. In some cases, Cd bioaccumulates in edible plant parts which comprise risk to consumers, because of Cd is a harmful heavy metal that can cause potent environmental and health hazards. For instance, Cd enrichment of cacao seeds have led to Cd enrichment of cacao-based products. In Latin America and the Caribbean, Cd bioaccumulation in cacao seeds occurs in different regions with diverse edaphoclimatic conditions, which makes it difficult to select soil remediation alternatives. Limited resources require that potential amendments must be carefully investigated through laboratory and/or greenhouse conditions before scaling up to field experiments. In this study, we evaluated the effectiveness of four biochars: coffee-, quinoa-, and inoculated- and palm-biochar, derived from three feedstocks: coffee husk, quinoa straw, and oil palm residues, respectively. Biochars were applied in two rates (1 and 2% w/w) in two soils, one moderately acidic and one slightly alkaline (Cd-spiked and non-spiked). CCN-51 cacao plants were used for the greenhouse experiment. After 130 days, biometric parameters, the bioavailability of Cd in the soil, and the concentration of Cd and mineral nutrients in the plants were measured. Quinoa biochar at the 2% significantly decreased (*P* < 0.01), by ∼71%, bioavailable Cd in moderately acidic and slightly alkaline soils, and leaf-Cd by ∼48%. Soil pH, electrical conductivity, and effective cation exchange capacity were significantly (*P* < 0.01) correlated with bioavailable soil and leaf-Cd. Biochar characteristics, such as ash contents, basic cations content, and surface functional groups could be used as indicators for the selection of biochars to reduce Cd uptake by cacao. Additionally, application of quinoa derived biochar provided P and K, which could increase productivity to offset mitigation costs. Overall, incorporation of quinoa biochar at 2% rate is effective for lowering bioavailable Cd in different soil types which reduces leaf-Cd in cacao plants.

## Highlights

• Biochar ash content helps to increase soil pH and reduce Cd bioavailability.
• Quinoa biochar at 2% reduced up to 80% bioavailable Cd in moderately acidic and slightly alkaline soils.
• Quinoa biochar at 2% lowered, up to 48%, Cd concentration in cacao plants.
• Soil pH, CECe, and EC were significant and negative related to bioavailable and plant-Cd.
• Ash content could be used as a guide for selecting biochars for Cd remediation.

## 1 Introduction

Cadmium (Cd) is a natural-occurring soil metal that can potentially affect human health (Smolders and Mertens, 2013). Chronic exposures of Cd can cause bone disease, endocrine disruption and has been shown to be a potential carcinogen (Pan et al., 2009; Suhani et al., 2021). Although there are different Cd exposure routes (such as inhalation and cigarette smoking), the intake of Cd-enriched food is the main Cd pathway for the nonsmoking population (WHO, 2011; European Food Safety Authority, 2012; Suhani et al., 2021). This phenomena have been reported in edible plant parts of several crops like: wheat grains (Rizwan et al., 2016), rice grains (Li et al., 2017), and cacao seeds (Argüello et al., 2019), the mainstay for the chocolate industry. Environmental authorities have established concentration limits for Cd in chocolate, cocoa powder, and derivatives. For instance, the European Union and the State of California (USA) have established limits between 0.1 - 0.8 and 0.4–0.96 mg kg^−1^ Cd in final product, respectively (European Commission, 2014; Maddela et al., 2020). These limits for Cd are related to the final product, not to the concentration in the cacao seeds, however traders commonly applied levels of Cd in the beans (Bravo et al., 2022). For cacao seeds, commonly applied threshold for export to the EU market is 0.6 mg kg^−1^, an unofficial industry threshold (Argüello et al., 2019; Vanderschueren et al., 2022). In Latin America and the Caribbean the Cd concentrations in cacao seeds averaged 0.9, 1.0, 2.5, and 4.0 mg kg^−1^ in Ecuador (Argüello et al., 2019), Peru (Arévalo-Gardini et al., 2017), Honduras (Gramlich et al., 2018), and Colombia (Bravo et al., 2022), respectively. These values are much higher than African countries in which Cd concentration averaged 0.3 mg kg^−1^ (Bertoldi et al., 2016). In this context, Cd concentration in cacao seeds is a menace for the sustainability of small-scale farmers (Meter et al., 2019). Cadmium in cacao seeds is strongly related to Cd concentration in soils, key soil properties (*e.g*. soil pH, soil organic matter, and P content) (Chavez et al., 2016b; Argüello et al., 2019; Bravo et al., 2022), and content of other elements like Zn (Argüello et al., 2019). Soil Zn content may favor or decrease Cd bioaccumulation depending on Zn bioavailability and Zn/Cd ratio, since the Cd transporters are nor specific, and the uptake of this element has been related to Zn transporters (Argüello et al., 2020).

Then, in-situ Cd immobilization in cacao-growing soils using amendments, has been proposed as a promising option (Chavez et al., 2016a; Argüello et al., 2019); nonetheless, this alternative has been poorly explored to date. A widely known amendment to control soil contamination is biochar (Brewer et al., 2011). The immobilization mechanisms and their effectiveness depend on several factors *e.g.* soil pH (Xiao et al., 2019) and material characteristics (ash, basic cation, and P content) (López et al., 2020; Houssou et al., 2022). Soil pH is among the most critical parameters influencing the effectiveness of biochar in Cd mitigation (Xiao et al., 2019). Uchimiya et al. (2011) found that cation exchange mechanism played a dominant role when biochar was used to remediate acidic soils. Likewise, the contribution of alkaline substances by biochars plays a vital role when biochar is applied to alkaline soils. However, less information was available for the method of improving Cd immobilization efficiency taking into account the pH of the targeted soil (Jia et al., 2022). Cacao production areas cover a wide diversity of soils with contrasting properties (*e.g.* acidic and neutral to alkaline soils) (Chavez et al., 2016a; Argüello et al., 2019), which represents a challenge for the selection of soil amendments to reduce plant-available soil Cd. There is little information about Cd mitigation in cacao-growing soils, especially in neutral to alkaline soils. Only one previous study evaluated the effect of biochar application on Cd uptake by cacao plants (Ramtahal et al., 2019).

In order to better understand the complex interplay between Cd, soil and biochar, in a broader scope, there is a need to conduct systematic studies evaluating different types of biochars at various rates under distinctive soil conditions. Developing criteria for selecting amendments for field studies based on the chemical properties of the material and/or soil is urgent. The aim of this work was to evaluate the effects of four biochars at two low rates (1 and 2% w/w) on the properties of two distinctive cacao-growing soils (slightly alkaline and moderately acidic) and their relationship with Cd bioavailability and uptake by cacao plants. Such research could help to alleviate excessive Cd bioaccumulation in cacao growing regions.

## 2 Materials and methods

### 2.1 Soil selection and sampling

For an easier follow-up and understanding of the methodology, an experimental overview can be found in Supplementary information (Figure S1). Soils were collected at two cacao farms where elevated bean Cd were reported (Argüello et al., 2019). Slightly alkaline soil (pH 7.30) was collected in Manabí province (Northeast) whereas moderately acidic soil (pH 5.52) was collected in Azuay province (Southeast). At these two cacao farms, Cd concentration in cacao bean exceeded the Cd threshold of 0.60 mg kg^−1^ (the European Union limit for Cd in chocolate), considered excessive for trading. A total of 500 kg of surface soil (0–15 cm) was collected at each farm by composite sampling (twelve subsamples within each farm). Soils were then air dried, disaggregated, sieved (2-mm mesh) and mixed to obtain a homogenous substrate. Soil pH and electrical conductivity (EC) were measured using 2:1 water:soil ratio. Soil organic matter (SOM) was determined using an elemental analyzer (Elementar Analyzer, Germany). Pseudo-total concentration of Cd and mineral nutrients were measured by Inductively Coupled Plasma Optical Emission Spectrometry (ICP-OES, Optima 5300 DV, PerkinElmer, USA) after digesting 0.25 g of soil sample with 10 mL of Aqua Regia (1:3 HCl:HNO_3_) at 90 °C for 1 h and 140 °C for 3 h.

### 2.2 Biochars

The four biochars used in this study were produced by Bioenergía de los Andes (Ecuador) using a prototype of a modular auger reactor for the pyrolysis process, integrated with a combustion reactor (Heredia-Salgado et al., 2020). Biochars were produced from three feedstocks: coffee husk (Coffee-BC, 550 °C), oil palm residue (Inoculated-BC, 600 °C and Palm-BC, 600 °C), and quinoa straw (Quinoa-BC, 550 °C). Inoculated-BC is a commercial mixture of oil palm residue biochar with a microbiology consortium. More details about the production of these biochars were reported in López et al. (2020). Biochar pH and EC were determined using 1:10 soil to water ratio after 60 min. Ash content was determined by loss on ignition. Carbon (C) and nitrogen (N) content were determined using an elemental analyzer. Pseudo-total concentration of Cd and mineral nutrients in extracts were measured by using ICP-OES as indicated above. Fourier-transform infrared spectroscopy analysis (FTIR) was used to characterize the biochar active sites, for which an infrared spectrometer (PerkinElmer, model spectrum Two V10.4.2) equipped with an Attenuated Total Reflection (ATR) accessory (PerkinElmer) was employed, operating in the spectral range 400–4000 cm^−1^ with a resolution of 4 cm^−1^.

### 2.3 Soil preparation and incorporation of biochars

Before biochar incorporation, the moisture contents of soils were kept at 40% water holding capacity (WHC) with deionized water (DW). Then, each biochar was incorporated in the soils at two rates (1 and 2% dry w/w) and soils without biochar were kept as controls. Later, soil moisture was adjusted to 80% of WHC with DW and equilibrated for 7 days. After equilibrium, the soils with and without biochars were divided in two parts, one part was kept as it is in the field (natural Cd concentration) and the other part was spiked with Cd (enriched conditions) using 1.00 mg Cd [from Cd(NO_3_)_2_•4H_2_O] per kg of soil. The former to evaluate the effectiveness of biochars under “natural” conditions and the latter to determine the potential of biochars to mitigate “enriched” conditions. The Cd concentration in spike solution was selected based on total soil Cd reported in Argüello et al. (2019) and was calculated to include the 97.5 percentile of the soil Cd reported in Ecuador, approximately 1.40 mg kg^−1^. The spiking solution was spread onto the soils while mixing thoroughly using a plastic spatula. The pseudototal Cd concentration in slightly alkaline soil and moderately acidic soil were 0.57 (±0.06) and 0.61 (±0.12) mg kg^−1^, respectively. After spiking, average pseudototal Cd concentration in slightly alkaline soil and moderately acidic soil samples were 1.38 (±0.44) and 1.64 (±0.14) mg kg^−1^, respectively, reaching the targeted concentrations. All treatments were brought to 80% WHC with DW and allowed to further equilibrate for 8 days.

### 2.4 Greenhouse experiment

After the 8-day equilibrium, 4 kg of soil of each treatment was transferred into polyethylene bags (30 × 40 cm width and depth, respectively) and then 6-month cacao plants were transplanted into each bag. Plant material (*cv.* CCN-51) was propagated by rooted cutting to avoid the intrinsic genetic variability of seed propagation in cacao. 108 experimental units were arranged in a completely randomized design with three replicates. During the greenhouse experiments, plants were watered (with DW) periodically to maintain 80% WHC. Cacao plants were fertilized monthly using 1.76 g bag^−1^ of Multi K (13% N, 0% P, 14% K) and 0.44 g bag^−1^ of Rafos (12% N, 24% P, 12% K, 2% Mg). Plants were allowed to grow under greenhouse conditions, 12 h light and 12 h dark, and average day temperature of 30 ± 1 °C, for 130 days. During the greenhouse experiments soil subsamples were withdrawn from each bag at 15, 30, 60 and 90 days to determine the pH dynamics.

### 2.5 Plant harvest and analyses

After 130 days, stem length, root length and stem diameter, and shoot and root dry weight were measured. Cacao plants were separated into leaves, stem and roots and subsequently washed. Leaves and stem were washed with DW, whereas roots were washed with tap water and then with DW, and subsequently immersed in a 0.01 M EDTA solution for 5 min followed by rinsing with DW. Roots, stems and leaves were dried at 65 °C for 48 h and dry biomass was measured. Dried tissues were ground to determine elemental composition of each plant part. In brief, 300 mg of pulverized dry biomass were digested with 5.0 mL of HNO_3_ at 80 °C for 6 h. After digestion, samples were diluted with DW and passed through a 0.45 μm filter paper. Mineral nutrients and Cd in the filtrates were determined by ICP-OES.

### 2.6 Post-harvest soil samples and chemical analyses

After harvest, soil samples were collected and pH, EC, SOM, effective cation exchange capacity (CECe), and Cd pools were measured. Soil pH, EC, and SOM were also measured as indicated above for biochars. Soil CECe was determined as a sum of exchangeable base cations extracted with 1 M NH_4_OAc solution. Three different extraction procedures were used to study the Cd pools in soil samples. The extraction methods were selected based on their extraction power and targeted pools, which are explained as follows: 0.01 M CaCl_2_ extract water-soluble and readily exchangeable metals (CaCl_2_-Cd), 1 M NH_4_OAc extract soluble-exchangeable metals (NH_4_OAc-Cd), and 0.1 M HCl extract exchangeable and acid-soluble metals (HCl-Cd) (Adamo and Zampella, 2008). For the CaCl_2_-, NH_4_OAc-, and HCl-Cd extractions were using 1:5, 1:5, and 1:20 sample to extractant ratio and equilibration time of 2, 2, and 3 h, respectively. Cadmium in supernatants was determined by ICP-OES.

### 2.7 Quality assurance, quality control, and data analysis

To assure the reliability of the results, quality assurance and quality control protocols were included throughout the analytical processes (see Supplementary Description S1). Two-way ANOVA was used to compute interactions between biochar type and rate. To determine the main effect of biochar or rate, one-way ANOVA was employed. *Post hoc* tests (Tukey and Dunnet) were applied to calculate differences among groups. For all the analyses, statistical differences were set at 5 %. Multivariate analysis between soil properties, soil Cd pools, and leaf-Cd was used to understand the interactions between soils and biochars. All data was analyzed using JMP® Pro version 13.1.0 software. When necessary, the continuous variables were transformed to comply with statistical assumptions. Reduction factors (Rf) were calculated using the ratio between Cd concentration in control divided by the corresponding concentration in treated soil/plant. Rf aims to illustrate the proportional change caused by the treatment as compared to the control, i.e., values over 1 imply a reduction in Cd concentration whereas values lower than 1 the opposite (Vanderschueren et al., 2021).

## 3 Results

### 3.1 Soil and biochars properties

The physicochemical properties of the soils and biochars are listed in Table 1. The two soils presented contrasting characteristics, mainly pH. Biochar pH was similar among all the studied biochars. In contrast, properties such as EC, ash, basic cations, and carbon content, displayed greater variability. Carbon content was highest in Inoculated-BC (67.8 %) and lowest in Quinoa-BC (56.8 %) whereas biochar pH and mineral constituents (*e.g.* ash and basic elements) were highest in Quinoa-BC and Coffee-BC. The C/N ratio in Quinoa- and Coffee-BC was on average 40% lower compared to Palm- and Inoculated-BC. Table S1 shows the result of the FTIR analysis. A greater number of functional groups were detected on the surface of Quinoa-BC in comparison with Coffee-, Palm-, and Inoculated-BC. Pseudototal Cd concentration was below the limit of detection of ICP-OES in all the studied biochars.

**Table 1:** Soil and biochar properties. Values are averages of three replicates (n = 3) and ±SE represent the standard errors of the mean.

Soil properties	Slightly alkaline soil	Moderately acidic soil	Quinoa-BC	Coffee-BC	Palm-BC	Inoculated-BC
SOM (%)∗∗	3.8 ± 0.2	5.2 ± 0.2	-	-	-	-
pH	7.3 ± 0.1	5.5 ± 0.1	10.7 ± 0.0	10.2 ± 0.0	9.6 ± 0.1	9.5 ± 0.1
EC (dS m^−1^)∗∗∗	2.1 ± 0.2	1.7 ± 0.1	12.9 ± 0.3	10.1 ± 0.2	0.3 ± 0.0	0.3 ± 0.0
P (g kg^−1^)	0.6 ± 0.1	0.4 ± 0.0	2.6 ± 0.2	2.3 ± 0.0	0.3 ± 0.0	0.2 ± 0.0
K (g kg^−1^)	4.3 ± 0.1	0.8 ± 0.1	49.1 ± 1.9	46.6 ± 0.3	2.5 ± 0.1	2.5 ± 0.1
Ca (g kg^−1^)	7.4 ± 0.1	2.5 ± 0.1	11.2 ± 0.6	6.3 ± 0.1	0.6 ± 0.0	0.5 ± 0.1
Mg (g kg^−1^)	7.0 ± 0.2	2.2 ± 0.1	10.1 ± 0.4	1.6 ± 0.0	0.4 ± 0.0	0.3 ± 0.0
Na (g kg^−1^)	0.6 ± 0.1	0.1 ± 0.0	0.8 ± 0.2	0.1 ± 0.0	0.1 ± 0.0	0.1 ± 0.0
Cd (mg kg^−1^)	0.6 ± 0.1	0.6 ± 0.1	BLD	BLD	BLD	BLD
Ash (%)	-	-	26.3 ± 0.1	19.6 ± 0.1	20.9 ± 0.1	11.8 ± 0.3
C (%)	-	-	56.8	66.2	64.4	67.8
N (%)	-	-	1.1	2.2	1.0	0.9
C/N	-	-	49.8	30.1	63.7	72.1

∗BLD: Below the Limit of Detection. ∗∗SOM: soil organic matter. ∗∗∗ EC: electrical conductivity.

### 3.2 Soil properties and Cd pools

**Figure 1:**
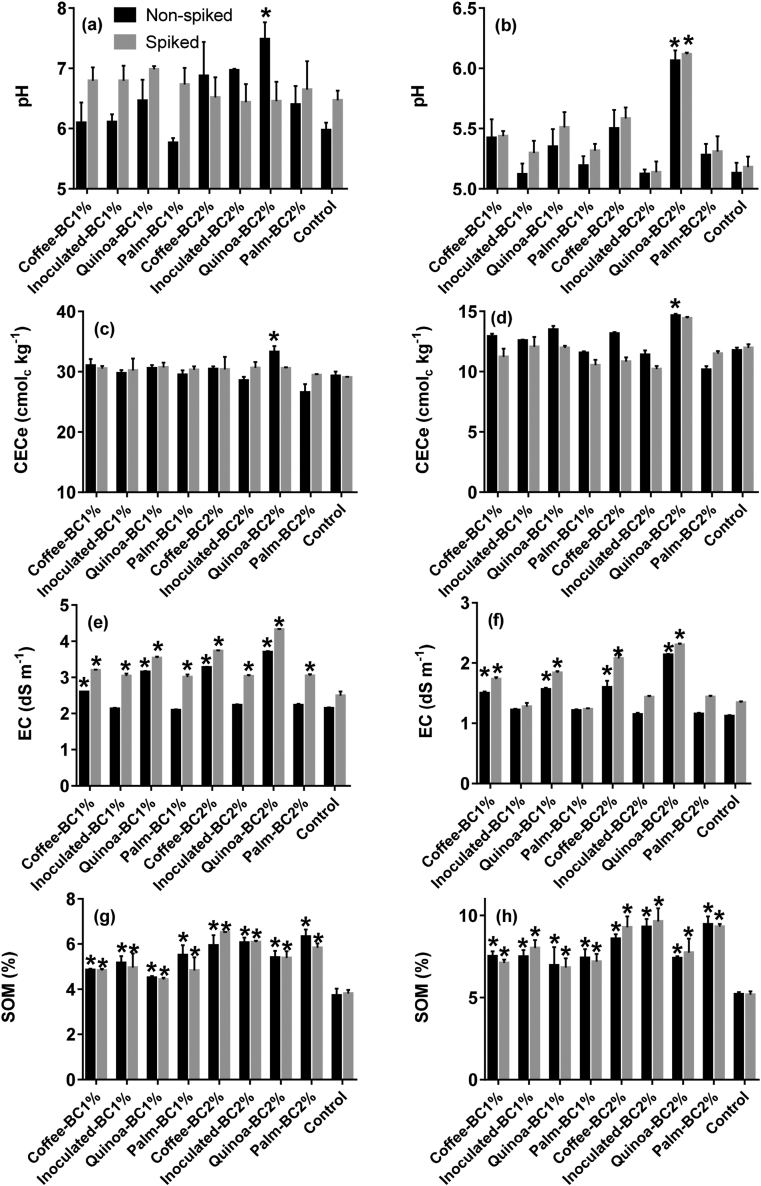
Changes on soil pH, effective cation exchange capacity (CECe), electrical conductivity (EC), and soil organic matter (SOM) in coffee-, inoculated-, quinoa-, and palm-biochar amended soils at 1 and 2% rate at 130 days after greenhouse experiment. Slightly alkaline soil (a, c, e and, g). Moderately acidic soil (b, d, f and, h). Error bars are standard errors (SE) of the means (n = 3). Asterisks above the bar indicate a significant (*P* < 0.05) difference between the treatment and the control by Dunnett's test.

The effect of biochar application and rate on soil pH is shown in Figure 1 ab, Figure S2 and Table S2. All biochars increased the soil pH, however, the liming effect was not maintained for a long period, i.e., >14 days (Figure S2). Only Quinoa-BC at 2% showed a consistent and more extended liming effect compared to Coffee-, Inoculated-, Palm-BC, and the control, regardless of soil type (Figure S2). Quinoa-BC at 2% significantly increased *(P <* 0.05) soil pH after 130 days by ∼1.11 units and ∼0.90 units in slightly alkaline and moderately acidic soils, respectively. Quinoa-BC at 2% raised soil pH by 1.51 in non-spiked slightly alkaline soil (Figure 1a), 0.85 in non-spiked moderately acidic and 0.93 in Cd-spiked moderately acidic soils (Figure 1b). No statistical difference was detected in soil pH in Cd-spiked slightly alkaline soils (Figure 1b).

The effect of biochar application and rate on the CECe is shown in Figure 1(c and d) and Table S2. In line with the pH trend, CECe was higher in biochar-amended soils. Quinoa-BC at the 2% rate increased CECe by 13 % in non-spiked slightly alkaline soil, 11 % in non-spiked moderately acidic soil (Figure 1d), and 16 % in Cd-spiked moderately acidic soil (Figure 1d). There was no statistical effect (*P* > 0.05) in the application of biochars in Cd-spiked slightly alkaline soils (Figure 1c). Soil EC increased proportionally with application rate (Figure 1 [e and f] and Table S2). Application of Coffee- and Quinoa-BC showed the most significant (*P* < 0.05) increases in soil EC in all soils with ∼27.7% higher EC in slightly alkaline soil with (Figure 1e) and ∼26.1% greater EC in moderately acidic soils (Figure 1f). Cd-spiked slightly alkaline soil showed a significant increment in EC (*P* < 0.05) for all biochar-amended soils (Figure 1e). In Cd-spiked moderately acidic soil this phenomenon was not observed.

The application of biochars significantly increased (*P* < 0.05) SOM regardless of soil type or Cd treatment (Figure 1gh and Table S2). On average, SOM increase was in line with the application rate, which is due to the organic carbon inputs from biochar (Table 1). Our results showed SOM increased between 18 and 77% in slightly alkaline soil (Figure 1g) and between 31 and 82% in moderately acidic soil (Figure 1h).

Cadmium pools in soils were determined at 130 days using three single-step extraction methods as shown in Table 2. Water-soluble and easily exchangeable Cd (CaCl_2_-Cd) and exchangeable Cd (NH_4_OAc-Cd) represent the directly bioavailable and reactive pools of Cd in the soil, respectively, while HCl-Cd represents the least available pool (Pan et al., 2016). Quinoa-BC at 2% significantly decreased soluble and exchangeable Cd pools in soils by ∼71% (Table 2). Effects of biochar application and rate on reduction factor (Rf) of soil Cd pools are shown in Table 3 and Table S3. Increasing the biochar rate significantly reduced (*P* < 0.05) CaCl_2_- and NH_4_OAc-Cd. The largest Rf in CaCl_2_-Cd was observed with application of Quinoa-BC at 2% for all soils, under basal and Cd-enriched conditions. The Rf in soils treated with Quinoa-BC at 2% was ∼4-fold higher than the other biochars. CaCl_2_-Cd concentration in non-spiked slightly alkaline soils were lower than the detection limit of the ICP-OES and therefore are not reported. Similarly, NH_4_OAc-Cd was significantly (*P* < 0.05) reduced by the application of Quinoa-BC at 2%. The corresponding Rf for this pool was ∼2-fold higher for all slightly alkaline and moderately acidic soils. HCl-Cd was reduced only in Cd-spiked moderately acidic soil with the application of Quinoa-BC.

**Table 2:** Leaf-Cd concentration and CaCl_2_-, NH_4_OAc-, and HCl-Cd concentration in non-spiked and Cd-spiked slightly alkaline and moderately acidic soils amended with coffee-, inoculated-, quinoa-, and palm-biochar at 1 and 2% rate at 130 days after greenhouse experiment. Values are averages of three replicates (n = 3) and ±SE represent the standard errors of the mean. Different letters indicate statistical significance at *P* < 0.05 (Tukey's test) of the soil and Cd treatment group. Data of CaCl_2_-Cd for non-spiked slightly alkaline soil below the detection limit of equipment.

Soil		Treatment	CaCl_2_-Cd (μg kg^−1^)	NH_4_OAc-Cd (μg kg^−1^)	HCl-Cd (μg kg^−1^)	Leaf-Cd (mg kg^−1^)
Slightly alkaline	Non-spiked	Coffee-BC1%	-	85.0 ± 2.9 abc	446 ± 20 a	3.1 ± 0.4 a
		Coffee-BC2%	-	70.0 ± 2.9 cd	418 ± 8 a	2.4 ± 0.2 a
		Inoculated-BC1%	-	93.3 ± 1.7 ab	456 ± 24 a	2.8 ± 0.3 a
		Inoculated-BC2%	-	101 ± 7 a	431 ± 13 a	3.1 ± 0.6 a
		Quinoa-BC1%	-	75.0 ± 2.9 cd	431 ± 9 a	2.8 ± 0.1 a
		Quinoa-BC2%	-	60.0 ± 0.0 d	428 ± 10 a	1.8 ± 0.4 a
		Palm-BC1%	-	98.3 ± 1.7 ab	442 ± 14 a	2.4 ± 0.3 a
		Palm-BC2%	-	83.3 ± 6.0 bc	395 ± 18 a	2.4 ± 0.2 a
		Control	-	98.3 ± 3.3 ab	471 ± 17 a	2.9 ± 0.4 a
Slightly alkaline	Spiked	Coffee-BC1%	16.4 ± 2.4 a	165 ± 7.6 abc	749 ± 25 bc	5.2 ± 0.3 ab
		Coffee-BC2%	15.3 ± 0.2 ab	203 ± 15.9 a	731 ± 2 c	4.7 ± 0.3 ab
		Inoculated-BC1%	14.7 ± 0.6 ab	188 ± 6.0 ab	753 ± 26 abc	5.2 ± 0.4 ab
		Inoculated-BC2%	8.9 ± 1.1 bc	147 ± 8.8 bc	764 ± 15 abc	3.7 ± 0.4 b
		Quinoa-BC1%	10.0 ± 0.2 bc	153 ± 6.7 bc	783 ± 30 abc	4.5 ± 0.2 ab
		Quinoa-BC2%	4.9 ± 0.6 c	137 ± 3.3 c	801 ± 12 abc	3.9 ± 0.0 b
		Palm-BC1%	13.6 ± 0.4 ab	183 ± 15.9 ab	862 ± 35 a	5.9 ± 0.6 a
		Palm-BC2%	14.5 ± 0.2 ab	190 ± 3 ab	844 ± 22 ab	4.3 ± 0.6 ab
		Control	19.7 ± 1.3 a	200 ± 5 a	828 ± 19 abc	4.6 ± 0.3 ab
Moderately acidic	Non-spiked	Coffee-BC1%	61.2 ± 5.5 ab	162 ± 3 abcd	510 ± 18 a	4.3 ± 0.4 ab
		Coffee-BC2%	59.5 ± 5.0 abc	132 ± 7 d	487 ± 10 a	3.7 ± 0.1 ab
		Inoculated-BC1%	67.2 ± 11.1 abc	185 ± 5 a	512 ± 13 a	4.8 ± 0.6 ab
		Inoculated-BC2%	57.0 ± 10.7 abc	168 ± 4 abc	493 ± 9 a	4.6 ± 0.5 ab
		Quinoa-BC1%	45.9 ± 3.9 abc	142 ± 6 cd	482 ± 9 a	3.7 ± 0.3 ab
		Quinoa-BC2%	22.9 ± 1.3 c	92.7 ± 10.9 e	519 ± 30 a	2.6 ± 0.1 b
		Palm-BC1%	45.3 ± 9.8 abc	180 ± 23 ab	539 ± 11 a	5.0 ± 0.1 ab
		Palm-BC2%	39.7 ± 9.1 bc	150 ± 10 bcd	489 ± 1 a	5.5 ± 0.8 a
		Control	80.1 ± 1.9 a	185 ± 3 a	529 ± 28 a	4.6 ± 1.0 ab
Moderately acidic	Spiked	Coffee-BC1%	151 ± 7 bc	412 ± 23 bcd	1471 ± 57 ab	13.2 ± 0.1 abc
		Coffee-BC2%	115 ± 2 bcd	338 ± 17 def	1342 ± 9 b	9.1 ± 0.4 bc
		Inoculated-BC1%	185 ± 7 ab	492 ± 33 ab	1508 ± 45 a	16.8 ± 1.5 a
		Inoculated-BC2%	191 ± 13 ab	467 ± 15 bc	1465 ± 19 ab	14.9 ± 2.1 ab
		Quinoa-BC1%	139 ± 16 bc	290 ± 22 f	870 ± 19 c	12.6 ± 1.4 abc
		Quinoa-BC2%	54.3 ± 14 d	298 ± 8 f	857 ± 10 c	6.4 ± 0.6 c
		Palm-BC1%	126 ± 16 bcd	392 ± 12 cde	1401 ± 6 ab	8.1 ± 1.7 bc
		Palm-BC2%	83.4 ± 3.8 cd	318 ± 3 def	1380 ± 46 ab	9.7 ± 2.1 bc
		Control	246 ± 38 a	568 ± 12 a	1454 ± 19 ab	13,9 ± 1.1 ab

**Table 3:** CaCl_2_-, NH_4_OAc-, HCl-Cd reduction factor (Rf) values in non-spiked and Cd-spiked slightly alkaline and moderately acidic soils amended with coffee-, inoculated-, quinoa-, and palm-biochar at 1 and 2% rate at 130 days after greenhouse experiment. Values are averages of three replicates (n = 3) and ±SE represent the standard errors of the mean. Different letters indicate statistical significance at *P* < 0.05 (Tukey's test). Data of CaCl_2_-Cd for non-spiked slightly alkaline soil below the detection limit of equipment.

Soil		Treatment	Rf CaCl_2_-Cd	Rf NH_4_OAc-Cd	Rf HCl-Cd
Slightly alkaline	Non-spiked	Coffee-BC1%	-	1.2 ± 0.0 cd	1.1 ± 0.1 a
		Coffee-BC2%	-	1.4 ± 0.0 b	1.1 ± 0.0 a
		Inoculated-BC1%	-	1.1 ± 0.0 d	1.0 ± 0.1 a
		Inoculated-BC2%	-	1.0 ± 0.0 d	1.1 ± 0.0 a
		Quinoa-BC1%	-	1.3 ± 0.1 bc	1.1 ± 0.0 a
		Quinoa-BC2%	-	1.6 ± 0.0 a	1.1 ± 0.0 a
		Palm-BC1%	-	1.0 ± 0.0 d	1.1 ± 0.0 a
		Palm-BC2%	-	1.2 ± 0.1 bcd	1.2 ± 0.1 a
Slightly alkaline	Spiked	Coffee-BC1%	1.2 ± 0.2 c	1.2 ± 0.1 abcd	1.1 ± 0.0 ab
		Coffee-BC2%	1.3 ± 0.0 c	1.0 ± 0.1 d	1.1 ± 0.0 a
		Inoculated-BC1%	1.4 ± 0.1 c	1.1 ± 0.0 cd	1.1 ± 0.0 ab
		Inoculated-BC2%	2.2 ± 0.3 b	1.4 ± 0.1 ab	1.1 ± 0.0 ab
		Quinoa-BC1%	2.0 ± 0.0 bc	1.3 ± 0.1 abc	1.1 ± 0.0 ab
		Quinoa-BC2%	4.1 ± 0.5 a	1.5 ± 0.0 a	1.0 ± 0.0 ab
		Palm-BC1%	1.5 ± 0.1 bc	1.1 ± 0.1 bcd	0.9 ± 0.0 b
		Palm-BC2%	1.4 ± 0.0 c	1.0 ± 0.0 cd	1.0 ± 0.0 b
Moderately acidic	Non-spiked	Coffee-BC1%	1.3 ± 0.1 b	1.1 ± 0.0 b	1.0 ± 0.0 a
		Coffee-BC2%	1.4 ± 0.1 b	1.4 ± 0.1 b	1.1 ± 0.0 a
		Inoculated-BC1%	1.3 ± 0.2 b	1.0 ± 0.0 b	1.0 ± 0.0 a
		Inoculated-BC2%	1.5 ± 0.4 b	1.1 ± 0.0 b	1.1 ± 0.0 a
		Quinoa-BC1%	1.8 ± 0.1 b	1.3 ± 0.1 b	1.1 ± 0.0 a
		Quinoa-BC2%	3.5 ± 0.2 a	2.1 ± 0.3 a	1.0 ± 0.1 a
		Palm-BC1%	1.9 ± 0.4 b	1.0 ± 0.0 b	1.0 ± 0.0 a
		Palm-BC2%	2.2 ± 0.4 b	1.2 ± 0.1 b	1.1 ± 0.0 a
Moderately acidic	Spiked	Coffee-BC1%	1.6 ± 0.1 bc	1.4 ± 0.1 de	1.0 ± 0.0 b
		Coffee-BC2%	2.1 ± 0.0 b	1.7 ± 0.1 bcd	1.1 ± 0.0 b
		Inoculated-BC1%	1.3 ± 0.0 c	1.2 ± 0.1 e	1.0 ± 0.0 b
		Inoculated-BC2%	1.3 ± 0.1 c	1.2 ± 0.0 e	1.0 ± 0.0 b
		Quinoa-BC1%	2.0 ± 0.0 b	2.1 ± 0.1 a	1.7 ± 0.0 a
		Quinoa-BC2%	3.7 ± 0.3 a	1.9 ± 0.1 ab	1.7 ± 0.0 a
		Palm-BC1%	2.2 ± 0.2 b	1.5 ± 0.0 cde	1.0 ± 0.0 b
		Palm-BC2%	3.1 ± 0.1 a	1.8 ± 0.0 abc	1.0 ± 0.0 b

### 3.3 Leaf-Cd content

**Figure 2:**
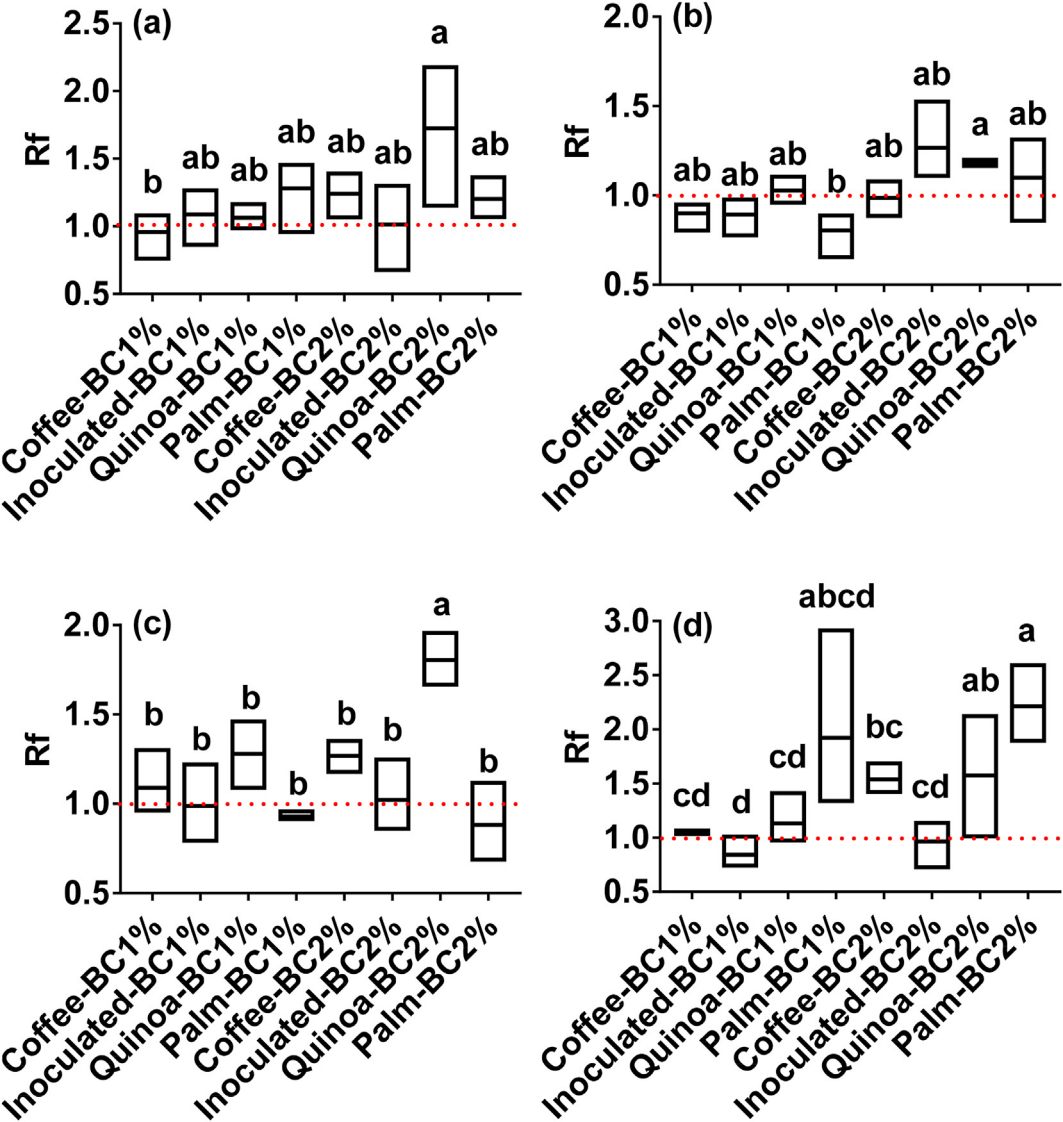
Box plot for reduction factor (Rf) in coffee-, inoculated-, quinoa-, and palm-biochar amended soils at 1 and 2% rate at 130 days after greenhouse experiment. Non-spiked (a) and Cd-spiked (b) slightly alkaline soils. Non-spiked (c) and Cd-spiked (d) moderately acidic soils. Red line represents 1, values greater than 1 have a positive effect, i.e., lower Cd in plants, whereas values lower than 1 is the opposite (Vanderschueren et al., 2021). Values represent the mean (n = 3). Different letters indicate statistical significance at *P* < 0.05 (Tukey's test).

**Figure 3:**
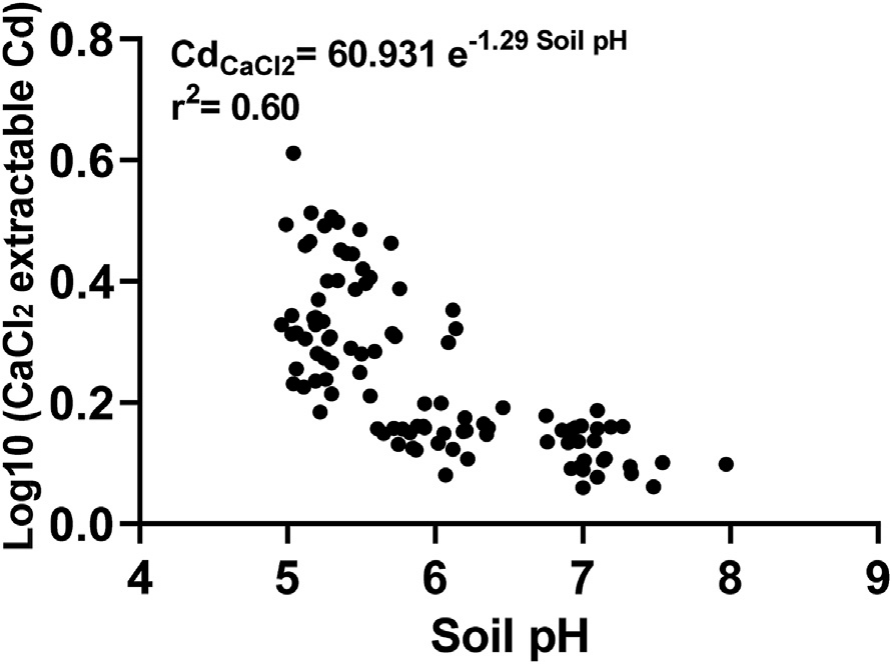
Relationships between soil pH and CaCl_2_ extractable Cd. The data corresponds to the soil pH of coffee-, inoculated-, quinoa-, and palm-biochar amended soils at 1 and 2% rate at 130 days after greenhouse experiment and values for CaCl_2_ extractable Cd. Data include values for non-spiked and Cd-spiked moderately acidic and Cd-spiked slightly alkaline soils. Figure does not take into account the CaCl_2_-Cd values of the non-spiked slightly alkaline soil.

Cadmium concentration in cacao leaves and the corresponding Rf for plant tissue are shown in Table 2 and Figure 2, respectively. Cadmium concentration in stems and roots are presented in Table S4. In general, cacao plants tend to compartmentalize Cd into the aerial parts. In line with the bioavailable Cd pools in soils, Cd concentration in cacao leaves was ∼1.4-fold higher in non-spiked moderately acidic soil compared with non-spiked slightly alkaline soil, whereas in Cd-spiked moderately acidic soil the value was ∼2-fold higher compared with Cd-spiked slightly alkaline soil. Overall, CaCl_2_-Cd and NH_4_AOc-Cd best represented the bioavailable fractions since they were best correlated with leaf-Cd (Figure 3). Effects of biochar application and rate on leaf-Cd concentration and Rf are presented in Table S5, Table S6, Table 2, and Figure 2. The application of Quinoa-BC at 2% significantly (*P* < 0.05) lowered Cd concentration in leaves in moderately acidic soil, Cd-spiked moderately acidic soil, and Cd-spiked slightly alkaline soil. In contrast, Cd in leaves in non-spiked slightly alkaline soils did not differ statistically but a lower concentration in the Quinoa-BC at 2% treatment was observed (Table 2).

To clearly show the impact of biochar application on leaf-Cd, the Rf was calculated (Figure 2 and Table S6). In line with leaf-Cd concentration, Quinoa-BC at 2% rate increased (*P* < 0.05) Rf in all treatments. Average Rf values in Quinoa-BC treatment (at the 2% rate) were 2.01 and 1.18 in non-spiked and Cd-spiked slightly alkaline soils, respectively. The corresponding Rf values for non-spiked and Cd-spiked moderately acidic soils were 1.80 and 2.20, respectively. In addition, with the Cd-spiked moderately acidic soil, Palm-BC at 2% showed similar Rf as Quinoa-BC.

### 3.4 Plant growth and mineral nutrition

No significant effects were found for the biometric parameters measured in the plants under the greenhouse conditions evaluated (*P* > 0.05, Table S8). The application of Coffee-, Quinoa-, and Palm-BC increased (*P* < 0.05) plant-P by at least 44% and plant-K over 31% in the moderately acidic soil (Table S8) (P and K deficient soil, Table 1). Similarly, Quinoa-BC at 2% increased plant-K by over 20 % in the slightly alkaline soil.

## 4 Discussion

### 4.1 Biochar properties varied according to feedstock materials

The variability found in the properties of biochars is explained by the different feedstocks and production conditions (López et al., 2020). The particular characteristics of each biochar suggest that their effect as a soil amendment could be variable, for instance can impact the ability of these materials for Cd immobilization (López et al., 2020). Higher C/N values of Palm- and Inoculated-BC indicate greater aromatic compounds compared to Quinoa- and Coffee-BC. This suggests that the latter two may have a higher Cd adsorption capacity. Low aromaticity provides more sorption sites due to cation (Cd) interaction with existing π-electronic systems from C=C bounds of the aromatic structure of the biochar (López et al., 2020). The oxygen-containing functional groups (hydroxyl, carboxylate, and phenolic hydroxyl) detected in Quinoa-BC could play an important role in the Cd adsorption via Cd-ligands coordinations bonds (López et al., 2020). On the other hand, a higher ash content is related to a higher alkalinity of the materials (Yuan et al., 2011), which could affect soil pH in the long term such as a significant increase in pH for acidic soils. Moreover, a higher content of basic cations indicates that Quinoa-BC could exchange these alkali elements with Cd in solution thus decreasing bioavailable Cd (Gao et al., 2019; López et al., 2020).

Due to the complex nature of Cd chemistry in soils, identifying the mechanisms that affectively bind Cd to biochar to prevent its uptake by cacao plant is an important task. In a previous study we found that one of the main mechanisms for competitive binding from the soil to the plant are mostly due to ion exchange with surface cations and precipitation as insoluble matter (López et al., 2020), which are consistent with the strong dependencies of the Cd absorption with the soil pH (Smolders and Mertens, 2013).

The results revealed that chemical properties of the soils were significantly affected by the addition of biochars, especially Quinoa-BC. The higher alkali nature, ash and basic cations content, and superficial functional groups of Quinoa-BC (Table 1 and Table S1) may explain its superior behavior to raise and maintain the soil pH comparing to the other biochars. Previous reports have related the liming effect of biochars with the presence of basic cations (Ca^2+^, Mg^2+^, K^+^, and Na^+^), carbonates (CO^2-^_3_ and HCO^-^_3_) and soluble organic compounds (RCOO- and RO-) (Fidel et al., 2017). In our study, we observed a higher increase in soil pH with the incorporation of Quinoa-BC at 2%. With similar application rates, researchers reported increments of 0.05 and 0.25 units in alkaline soils (Azhar et al., 2019; Xiao et al., 2019) and 0.24 and 1.08 units in acidic soils (Houben et al., 2013; Shen et al., 2016). This confirms a strong and prolonged liming power of Quinoa-BC. For the Cd-spiked alkaline soils, a stable pH behavior (Figure 1b) could be due to the buffering of the alkaline materials, which is supported by the significant (*P* < 0.05) increase in EC for the Cd-spike alkaline soil (Figure 1e) (Gao et al., 2019).

Application of biochars increased SOM due to the organic carbon inputs from biochar (Shi et al., 2020). We found a 10-fold increase in SOM in moderately acidic soil as compared to the slightly alkaline soil. The application of biochar into moderately acidic soil could ameliorate soil acidity and increase SOM by reducing CO_2_ release from soils (Bashir et al., 2018). Similarly, increases in SOM between 17 and 50% have been reported with the application of rice straw, rice hull, and maize stover biochars at application rates between 1.5 and 3% (Bashir et al., 2018).

The large increase in CECe by application of Quinoa-BC is explained by the raised in soil pH and SOM (Figure 1ab and 1gh, respectively), which enhances the negative soil surface charges and the surface functional groups present in the Quinoa-BC (Table S1) (Shi et al., 2017). Moreover, the concentration of basic cations in Quinoa-BC is higher compared to Coffee-, Inoculated-, and Palm-BC (Table 1), suggesting a greater cation exchange capacity (López et al., 2020). A higher EC was expected because these two biochars showed a higher content of basic cations as compared to Inoculated- and Palm-BC (Table 1). A release of weakly bound basic cations from the biochar matrix to the soil solution increases the EC values (Khan et al., 2017). The exchange between basic cations and labile Cd^2+^ might explain such increase (Gao et al., 2019). In Cd-spiked moderately acidic soil this phenomenon was not observed probably because of the low base cations of this soil (Table 1).

### 4.2 Soil cadmium pools affected by the incorporation of biochars

Changes in soil chemical properties impacted Cd pools and therefore Cd bioavailability in both soils. Soil pH, CECe, and SOM are key properties governing available Cd in soils (Smolders and Mertens, 2013). Our results showed that, after biochar addition, Cd pools, i.e., 0.01 M CaCl_2_- and 1 M NH_4_OAc-extractable Cd, decreased due to the increase of key soil properties, i.e., soil pH (Table 4). We found a significant (*P* < 0.01) and a negative relationship between bioavailable pools with soil pH, CECe, exchangeable basic cations, and EC (Table 4). Higher soil pH increases the sorption capacity of Cd in amended soils. This is caused by the variable surface charge, especially in moderately acidic soils (Wang and Siu, 2006). According to our results, soil pH is a good predictor of bioavailable Cd (Figure 3). Based on Figure 3, a pH value equal to or higher than 6.0 significantly reduce (up to 80%) the concentration of bioavailable Cd. Thus, biochars capable of increasing soil pH above this value may be good candidates for remediation in moderately acidic soils. A strong correlation between CaCl_2_-extractable Cd, CECe and basic cations (Table 4) supports our hypothesis that ion exchange was a major mechanism in the immobilization of Cd in soils amended with Quinoa-BC. Interestingly, there was significant (*P* < 0.01) positive correlation between SOM and both CaCl_2_- and NH_4_OAc-Cd (Table 4). This could be explained by the potential release of low weight organic molecules from biochars which could favor the formation of soluble organometallic complexes and increase soil-plant transfer (Hooda, 2010).

**Table 4:** Pearson's correlation analysis between soil properties and CaCl_2_-, NH_4_OAc-, and HCl-Cd. The data corresponds to the CaCl_2_-, NH_4_OAc-, and HCl-Cd values and soil properties of non-spiked and Cd-spiked moderately acidic soil and Cd-spiked slightly alkaline soil amended with coffee-, inoculated-, quinoa-, and palm-biochar at 1 and 2% rate at 130 days after greenhouse experiment. Statistical significance is represented by ∗*P* < 0.05, ∗∗*P* < 0.01. † NH_4_OAc-exchangeable basic cations.

	Cd-Leaves	CaCl_2_-Cd	NH_4_OAc-Cd	HCl-Cd	CECe	Ca†	K†	Mg†	Na†	SOM	pH	EC
Cd-Leaves		0.92∗∗	0.80∗∗	0.83∗∗	-0.62∗∗	-0.57∗∗	-0.61∗∗	-0.60∗∗	-0.56∗∗	0.40∗	-0.46∗	-0.30∗
CaCl_2_-Cd			0.84∗∗	0.63∗∗	-0.87∗∗	-0.83∗∗	-0.83∗∗	-0.86∗∗	-0.82∗∗	0.55∗∗	-0.78∗∗	-0,43∗
NH_4_OAc-Cd				0.90∗∗	-0.64∗∗	-0.58∗∗	-0.64∗∗	-0.61∗∗	-0.58∗∗	0.39∗	-0.49∗	-0.35∗
HCl-Cd					-0.39∗∗	-0.38∗	-0.35∗	-0.38∗	-0.37∗	0.23	-0.21	-0.08
CECe						0.99∗∗	0.91∗∗	0.99∗∗	0.92∗∗	-0.75∗∗	0.81∗∗	0.41∗
Ca†							0.85∗∗	0.98∗∗	0.91∗∗	-0.76∗∗	0.76∗∗	0.30∗
K†								0.89∗∗	0.83∗∗	-0.62∗∗	0,82∗∗	0.57∗∗
Mg†									0.95∗∗	-0.76∗∗	0.80∗∗	0.39∗
Na†										-0.72∗∗	0.78∗∗	0.46∗
SOM											-0.55∗∗	-0.16
pH												0.58∗∗
EC												

SOM: soil organic matter. CECe: effective cation exchange capacity. EC: electrical conductivity.

Previous studies have concluded that biochars with different feedstock and/or pyrolysis conditions are needed in order to reduce bioavailable Cd in acidic and alkaline soils. Qian et al. (2019) found that the immobilization of Cd and Zn in acidic and alkaline soils require the use of different biochars. Authors found that wheat biochar produced at low temperatures (350 °C) was more suitable for acidic soils, whereas biochar produced at high temperatures (650 °C) were more effective for alkaline soils. Biochar pH and phenolic functional groups were greater in low-temperature biochar which favor Cd immobilization in acidic soil. On the contrary, our results indicated that a biochar produced from quinoa straw, was effective in lowering available Cd in both soils. Uchimiya et al. (2011) found that different properties, such as cation exchange and alkaline substances, are key features in a biochar for its effective use in lowering bioavailable soil Cd.

Our findings suggest that the changes in soluble and exchangeable Cd in Quinoa-BC amended soils are related to direct and indirect immobilization mechanisms. Cadmium sorption could occur directly on the biochar particles by formation of complexes with functional groups present in the amendment (*e.g.* C-COOCd^+^ and C-OCd^+^) (Gong et al., 2021). In the other hand, as soil pH and CECe increases, Cd may be sorbed to variable-charge solids (Fe and Mn oxides) under formation of mononuclear, monodentate binding (*e.g.* ≡FeOH + Cd^2+^ -> ≡FeOHCd^+^ + H^+^) and by binuclear, bidentate binding (*e.g.* 2≡FeOH + Cd^2+^ -> ≡(FeO)_2_Cd + 2H^+^) (Borggaard et al., 2019). Additionally, the increase in SOM favors the formation of Cd-ligand complexation with surface functional groups like carboxylic, hydroxylic, and phenolic (*e.g.* Cd^2+^ + R–OH -> Cd-RO + H^+^) (Hamid et al., 2020). In the Cd-spiked moderately acidic soil, a significant change in HCl-extractable Cd may imply the formation of Cd-bearing minerals (Bashir et al., 2018). It was observed that high Cd concentration in solution favors the formation of Cd-bearing minerals, such as CdCO_3_. This occurs at the surface of the Quinoa-BC due to the reaction between Cd and carbonatious materials (López et al., 2020). Based on these results, properties such as a high base cation and ash content are desirable in biochars for the immobilization of Cd in cacao-growing soils. In addition, the capability of the biochar to maintain a high soil pH for a prolonged period of time is crucial. The latter is associated with the functional groups present in the material, which help to modify the soil buffering capacity (Shi et al., 2017). After the sorption of Cd in the biochar, this potentially toxic element continues in the soil, however, it will be in a less mobile form, reducing its bioavailability. Because immobilization in these materials occurs by mechanisms such as co-precipitation (López et al., 2020), Cd is less likely to return to the soil solution, but as mentioned above, this will be conditioned, for instance, by changes in soil pH. It is necessary to evaluate over time, how the Cd sorbed in the biochar could be exchanged in the soil solution, so reapplication of the biochar over time would be required.

### 4.3 Quinoa biochar reduced plant-Cd

**Figure 4:**
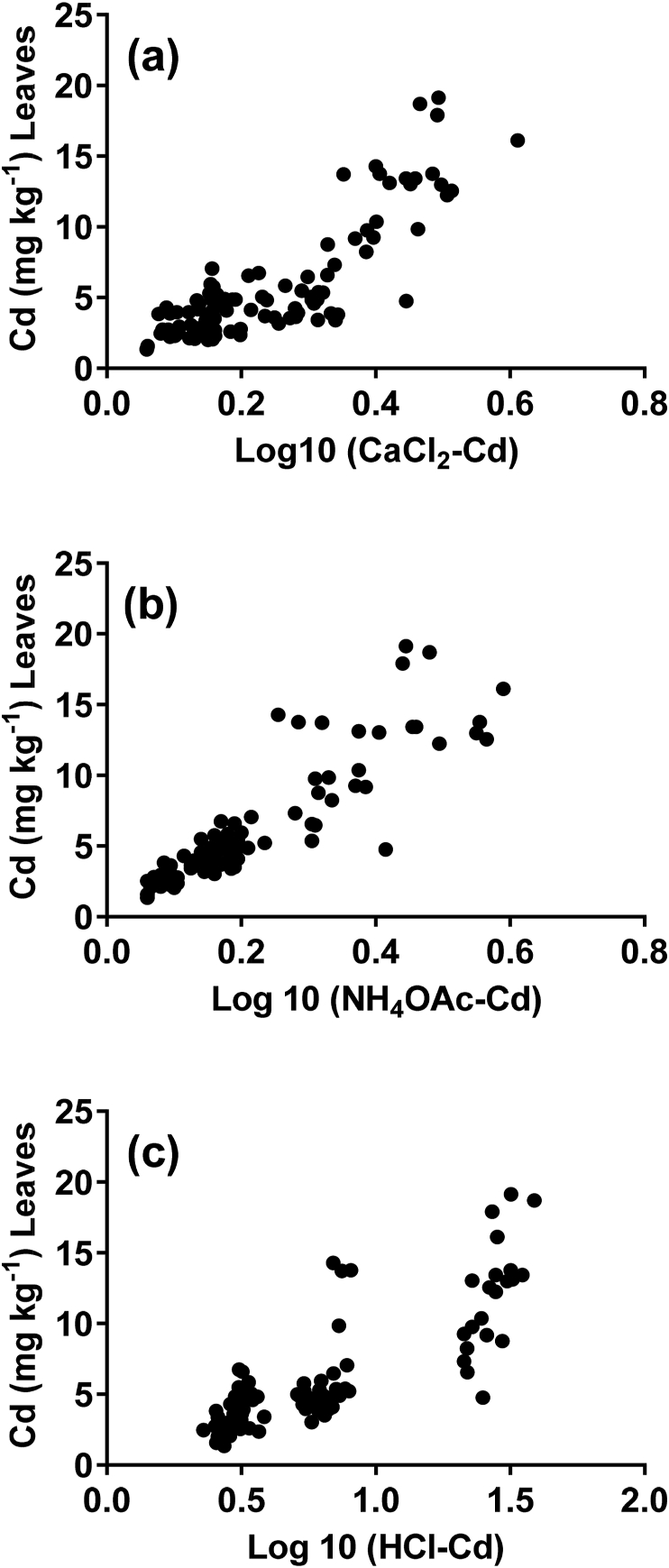
Leaf-Cd concentration as function of (a) CaCl_2_ extractable Cd (CaCl_2_-Cd), (b) NH_4_OAc extractable Cd (NH_4_OAc-Cd) and (c) HCl extractable Cd (HCl-Cd). The data corresponds to the CaCl_2_, NH_4_OAc, and HCl extractable Cd values and leaf-Cd concentration for plants of cacao cv CCN-51 planted on non-spiked and Cd-spiked moderately acidic soil and Cd-spiked slightly alkaline soil amended with coffee-, inoculated-, quinoa-, and palm-biochar at 1 and 2% rate at 130 days after greenhouse experiment. Figure does not take into account the CaCl_2_-Cd values of the non-spiked slightly alkaline soil.

A lower bioavailable soil Cd reduced the Cd concentration in cacao plants, specifically in the soil treated with Quinoa-BC at a 2% rate. Since cacao plant binds bioavailable Cd in the soil through the root, where TcNRAMP5 has been identified as an important transporter for Cd uptake from the soil solution (Ullah et al., 2018). Overall, CaCl_2_-Cd and NH_4_AOc-Cd best represented the bioavailable fractions since they were best correlated with leaf-Cd (Figure 4). These extraction procedures are simple to use and commonly apply to determine readily bioavailable Cd in soils (Adamo and Zampella, 2008).

Cadmium content in leaves was highly correlated with Cd concentration in roots and stems (Figure S3, r > 0.62, *P* < 0.01), which is explained by the fact that the cacao plant promotes some tolerance strategies such as uptake and transfer of Cd from the root to the shoot (Oliveira et al., 2022). It can be established that cacao is very efficient in translocating Cd to the leaves. Thus, avoiding the uptake of Cd by the root will decrease the concentration in stems and leaves. In addition, it has been reported that the differential Cd content in the cacao plant can be attributed to the preferential binding of Cd to compounds such as phytate, organic acids, and polyphenols (Vanderschueren et al., 2022).

The discussion will focus on leaves rather than stem or roots. Our results indicate that at a rate of 2%, Quinoa-BC can reduce the bioaccumulation of Cd in cacao leaves in both moderately acidic and slightly alkaline soils with varying concentrations of the contaminant. On average, leaf Cd was 1.8- and 2.5-fold lower when Quinoa-BC at 2% was applied on slightly alkaline and moderately acidic soils, respectively. For the acidic soil, the reduction of leaf Cd was higher than the one reported in Ramtahal et al. (2019) (2.5 vs 0.9-fold) with the application of commercial biochar (652 kg ha^−1^) after 120 days. Cadmium concentration in leaves were negatively correlated (*P* < 0.01) with soil properties, i.e., soil pH, CECe, and EC. The increase of soil pH in Quinoa-BC amended soils decreased bioavailable Cd, as discussed above, which consequently reduced Cd uptake in cacao plants. Aside from Quinoa, Only Palm-BC was able to reduce Cd bioaccumulation in leaves, but this was observed only in the Cd-spiked moderately acidic soil. In a batch experiment, with high-soluble Cd concentration, great Cd sorption onto the Palm-BC surface was observed (López et al., 2020). Thus, the Palm-BC at a 2% rate, in addition to Quinoa-BC, could potentially alleviate excessive bioavailable Cd in moderately acidic soils with total soil Cd higher than 1.60 mg kg^−1^. Cadmium concentration in leaves can be considered as a proxy for Cd bioaccumulation in cacao beans since these two tissues are highly correlated. For instance, correlation coefficients of 0.74 (Arévalo-Gardini et al., 2017) and 0.91 (Argüello et al., 2019) have been reported. Our results showed that Quinoa-BC effectively reduced leave-Cd in all soil conditions thus this amendment should be tested in field remediation trials.

To understand the effectiveness of biochars in lowering plant-Cd, the Rf values were computed. A Rf of 1.5 could have significant impact on cacao plantations affected by elevated Cd levels (Vanderschueren et al., 2021). With the application of Quinoa-BC an average Rf of 1.6 and 2.0 was observed in slightly alkaline and moderately acidic soil, respectively. In our study, the Rf found in moderately acidic soil is higher than the one found by Ramtahal et al. (2019) (2.0 vs 1.5) with the application of commercial biochar (652 kg ha^−1^) after 120 days. Azar et al. (2019) found a similar Rf (2.1) in wheat and rice plants using rice husk biochar at a 2% dose. A little higher Rf (3.7) was attained in a neutral-pH soil with the application of Miscanthus x giganteus biochar. To achieve such reduction, authors applied a much higher dose (10%) as compared to the one applied in our study. Indeed, at the lowest dose (1%), authors reported that Cd transfer was enhanced (Rf < 1) (Houben et al., 2013). This is in line with our findings, some biochars (*e.g*. Inoculated-BC) at low dose increased Cd transfer from soil to plants likely due to binding in low-weight organic molecules (O'Connor et al., 2018). Application of quinoa biochar at 2% reduced Cd uptake by plants by approximately 1.8-fold compared to the control. This reduction in the mass of Cd (uptake amount) implies a lower amount of contaminant entering the food chain, due to the mass of Cd immobilized in the biochar-amended soil.

Selecting a realistic rate is critical for studying the effect of biochar for Cd immobilization. It has been widely documented that high rates, *e.g*. 5% w:w, can reduce Cd transfer from soil to plant parts (Zhang et al., 2016; Dai et al., 2017). However, such application rates are not affordable under agricultural field conditions (Dai et al., 2017). In our study, Quinoa-BC at a realistic dose of to 40 t ha^−1^ (equivalent to 2%) showed to be a potential amendment to reduce Cd bioaccumulation in cacao plants in field studies. Biochar derived from quinoa has the characteristics to alter soils properties which reduces Cd accumulation in cacao plants.

Figure S4 shows the relationship between biochar ash, basic cations content, EC, and Rf of the studied biochars and other biochars reported in the literature (Houben et al., 2013; Mohamed et al., 2018; Xiao et al., 2019). The results indicated that these properties of biochars are significantly correlated (r = 0.66 to 0.85) with their effectiveness in reducing Cd bioaccumulation in plants. Likewise, Person's correlations showed a significant (P < 0.01) and negative relationship between biochar properties such as ash and basic cations content and CaCl_2_-, NH_4_OAc-, and HCl-Cd concentration (r = -0.50 to -0.96) (Table S7). Previous research reported that the ash content of biochars plays an important role in the sorption of metals (Wang et al., 2018; Yuan et al., 2019). In line with these results, biochars basic cations concentration showed to be a useful predictor of Cd immobilization in aqueous solutions (López et al., 2020). The characteristics of the biochars are critical for selecting an amendment suitable for field trials. It is suggested that ash content could be used as a guideline in the identification of potential biochar for Cd immobilization in soils.

### 4.4 Biochar application enhances nutrient levels in cacao plants

Aside from potential toxic elements (*e.g.* Cd) immobilization, biochars could improve soil fertility and plant nutrition (El-Naggar et al., 2019). The uptake of these essential plant nutrients can boost cacao yields. Remediation of Cd contaminated soils can be more sustainable if the amendments can provide essential plant nutrients and impact yields (Hamid et al., 2019). Our results indicated that the application of Quinoa-BC reduced plant-Cd and improved P and K uptake simultaneously (Table S8). Significant increase in plant-P and -K concentration in moderately acidic soil indicates the nutrient supply by the application of Quinoa-BC which could reduce the application rate of P and K fertilizers. Similarly, in slightly alkaline soils, an increase in plant-K was observed (Table S8). Similar increases in plant nutrients have been reported in other studies as a side effect of biochar application. Bashir et al. (2018) found that the addition of biochar at 3% significantly increased N, P and K concentration in Chinese cabbage (*Brassica rapa*). The application of bamboo chips and rice straw biochar at 2.5% significantly increased P and K content in *Brassica chinensis*. Remediation of Cd contaminated soils can be more sustainable if the suitable amendments can also provide plant nutrients and potentially increase yields (Hamid et al., 2019).

## 5 Conclusions

There is an urgent need to find agronomic countermeasures to alleviate Cd bioavailability in cacao growing areas. The incorporation of biochar derived from quinoa residues at a 2%, increased soil pH and effective cation exchange capacity which are key soil properties governing bioavailable Cd. Quinoa derived biochar display a lower bioavailable soil-Cd due to mechanisms such as co-precipitation and ion exchange decreasing leaf-Cd. Additionally, application of quinoa derived biochar provided plant mineral nutrients (P and K), which could increase productivity to offset mitigation costs. The selection of biochar based on its physicochemical properties, prior to field experiments, is essential to increase the success rate of Cd immobilization treatments on cacao farms. With our results it is possible to conclude that the ash and basic cations content, and functional groups could be good indicators for the selecting or engineer biochars to reduce Cd bioavailability in areas with diverse soil conditions. The results can be applied as a basis for the selection of materials for trials on cacao farms. A field experiment with quinoa derived biochar at a comparative rate, i.e., 40 Mg ha^−1^, should be implemented to evaluate the findings.

## Declarations

### Author contribution statement

Julián E. López: Conceived and designed the experiments; Performed the experiments, analyzed and interpreted the data; Wrote the paper.

Catalina Arroyave & Adriana Aristizábal: Conceived and designed the experiments; Analyzed and interpreted the data.

Byrone Almeida & Santiago Builes: Analyzed and interpreted the data.

Eduardo Chavez: Conceived and designed the experiments; Analyzed and interpreted the data; Contributed reagents, materials, analysis tools or data.

### Funding statement

Dr Eduardo Chavez was supported by 10.13039/501100000780European Union and implemented by CEFA/GIZ [FOOD/2016/380-060].

Dr Eduardo Chavez was supported by Research Project UNEMI [OCAS-02-2016].

Catalina Arroyave was supported by Patrimonio Autónomo Fondo Nacional de Financiamiento para la Ciencia, la Tecnología y la Innovación Francisco José de Caldas [120680863411].

Julián E. López was supported by Scholarship [201706101].

### Data availability statement

Data included in article/supp. material/referenced in article.

### Declaration of interest's statement

The authors declare no conflict of interest.

### Additional information

No additional information is available for this paper.

## References

[bib1] Adamo P., Zampella M. (2008). Environmental Geochemistry.

[bib2] Arévalo-Gardini E., Arévalo-Hernández C.O., Baligar V.C., He Z.L. (2017). Heavy metal accumulation in leaves and beans of cacao (*Theobroma cacao* L.) in major cacao growing regions in Peru. Sci. Total Environ..

[bib3] Argüello D., Chavez E., Lauryssen F., Vanderschueren R., Smolders E., Montalvo D. (2019). Soil properties and agronomic factors affecting cadmium concentrations in cacao beans: a nationwide survey in Ecuador. Sci. Total Environ..

[bib4] Argüello D., Montalvo D., Blommaert H., Chavez E., Smolders E. (2020). Surface soil liming reduces cadmium uptake in cacao (*Theobroma cacao* L.) seedlings but is counteracted by enhanced subsurface Cd uptake. J. Environ. Qual..

[bib5] Azhar M., Zia ur Rehman M., Ali S., Qayyum M.F., Naeem A., Ayub M.A., Anwar ul Haq M., Iqbal A., Rizwan M. (2019). Comparative effectiveness of different biochars and conventional organic materials on growth, photosynthesis and cadmium accumulation in cereals. Chemosphere.

[bib6] Bashir S., Hussain Q., Shaaban M., Hu H. (2018). Efficiency and surface characterization of different plant derived biochar for cadmium (Cd) mobility, bioaccessibility and bioavailability to Chinese cabbage in highly contaminated soil. Chemosphere.

[bib7] Bertoldi D., Barbero A., Camin F., Caligiani A., Larcher R. (2016). Multielemental fingerprinting and geographic traceability of Theobroma cacao beans and cocoa products. Food Control.

[bib8] Borggaard O.K., Holm P.E., Strobel B.W. (2019). Potential of dissolved organic matter (DOM) to extract As, Cd, Co, Cr, Cu, Ni, Pb and Zn from polluted soils: a review. Geoderma.

[bib9] Bravo D., Santander M., Rodríguez J., Escobar S., Atkinson Ramtahai G. (2022). `From soil to chocolate bar`: identifying critical steps in the journey of cadmium in a Colombia cacao plantation. Food Addit. Contam..

[bib10] Brewer C.E., Unger R., Schmidt-Rohr K., Brown R.C. (2011). Criteria to select biochars for field studies based on biochar chemical properties. BioEnergy Res.

[bib11] Chavez E., He Z.L., Stoffella P.J., Mylavarapu R., Li Y., Baligar V.C. (2016). Evaluation of soil amendments as a remediation alternative for cadmium-contaminated soils under cacao plantations. Environ. Sci. Pollut. Res..

[bib12] Chavez E., He Z.L., Stoffella P.J., Mylavarapu R.S., Li Y.C., Baligar V.C. (2016). Chemical speciation of cadmium: an approach to evaluate plant-available cadmium in Ecuadorian soils under cacao production. Chemosphere.

[bib13] Dai Z., Zhang X., Tang C., Muhammad N., Wu J., Brookes P.C., Xu J. (2017). Potential role of biochars in decreasing soil acidification - a critical review. Sci. Total Environ..

[bib14] El-Naggar A., El-Naggar A.H., Shaheen S.M., Sarkar B., Chang S.X., Tsang D.C.W., Rinklebe J., Ok Y.S. (2019). Biochar composition-dependent impacts on soil nutrient release, carbon mineralization, and potential environmental risk: a review. J. Environ. Manag..

[bib15] European Commission (2014). Commission Regulation (EU) No 488/2014 of 12 May 2014 amending Regulation (EC) No 1881/2006 as regards maximum levels of Cd in foodstuffs. Off. J. Eur. Union.

[bib16] European Food Safety Authority (2012). Cadmium dietary exposure in the European population: cadmium dietary exposure in Europe. EFSA J..

[bib17] Fidel R.B., Laird D.A., Thompson M.L., Lawrinenko M. (2017). Characterization and quantification of biochar alkalinity. Chemosphere.

[bib18] Gao X., Peng Y., Zhou Y., Adeel M., Chen Q. (2019). Effects of magnesium ferrite biochar on the cadmium passivation in acidic soil and bioavailability for packoi (*Brassica chinensis* L.). J. Environ. Manag..

[bib19] Gong H., Tan Z., Huang K., Zhou Y., Yu J., Huang Q. (2021). Mechanism of cadmium removal from soil by silicate composite biochar and its recycling. J. Hazard Mater..

[bib20] Gramlich A., Tandy S., Gauggel C., López M., Perla D., Gonzalez V., Schulin R. (2018). Soil cadmium uptake by cocoa in Honduras. Sci. Total Environ..

[bib21] Hamid Y., Tang L., Sohail M.I., Cao X., Hussain B., Aziz M.Z., Usman M., He Z., Yang X. (2019). An explanation of soil amendments to reduce cadmium phytoavailability and transfer to food chain. Sci. Total Environ..

[bib22] Hamid Y., Tang L., Hussain B., Usman M., Lin Q., Rashid M.S., He Z., Yang X. (2020). Organic soil additives for the remediation of cadmium contaminated soils and their impact on the soil-plant system: a review. Sci. Total Environ..

[bib23] Heredia-Salgado M.A., Coba S J.A., Tarelho L.A.C. (2020). Simultaneous production of biochar and thermal energy using palm oil residual biomass as feedstock in an auto-thermal prototype reactor. J. Clean. Prod..

[bib24] Hooda P.S. (2010). Trace Elements in Soils.

[bib25] Houben D., Evrard L., Sonnet P. (2013). Mobility, bioavailability and pH-dependent leaching of cadmium, zinc and lead in a contaminated soil amended with biochar. Chemosphere.

[bib26] Houssou A.A., Jeyakumar P., Niazi N.K. (2022). Biochar and soil properties limit the phytoavailability of lead and cadmium by *Brassica chinensis* L. in contaminated soils. Biochar.

[bib27] Jia Y., Li J., Zeng X., Zhang N., Wen J., Liu J., Jiku M.A., Wu C., Su S. (2022). The performance and mechanism of cadmium availability mitigation by biochars differ among soils with different pH: Hints for the reasonable choice of passivators. J. Environ. Manag..

[bib28] Khan W.D., Ramzani P.M.A., Anjum S., Abbas F., Iqbal M., Yasar A., Ihsan M.Z., Anwar M.N., Baqar M., Tauqeer H.M., Virk Z.A., Khan S.A. (2017). Potential of miscanthus biochar to improve sandy soil health, in situ nickel immobilization in soil and nutritional quality of spinach. Chemosphere.

[bib29] Li H., Luo N., Li Y.W., Cai Q.Y., Li H.Y., Mo C.H., Wong M.H. (2017). Cadmium in rice: transport mechanisms, influencing factors, and minimizing measures. Environ. Pollut..

[bib30] López J.E., Builes S., Heredia Salgado M.A., Tarelho L.A.C., Arroyave C., Aristizábal A., Chavez E. (2020). Adsorption of cadmium using biochars produced from agro-residues. J. Phys. Chem. C.

[bib31] Maddela N.R., Kakarla D., Garc a L.C., Chakraborty S., Venkateswarlu K., Megharaj M. (2020). Cocoa-laden cadmium threatens human health and cacao economy: a critical view. Sci. Total Environ..

[bib32] Meter A., Atkinson R.J., Laliberte B. (2019). Cadmium in cacao from Latin America and the Caribbean: a review of research and potential mitigation solutions. Rome (Italy): Biovers. Internat..

[bib33] Mohamed I., Ali M., Ahmed N., Abbas M.H.H., Abdelsalam M., Azab A., Raleve D., Fang C. (2018). Cow manure-loaded biochar changes Cd fractionation and phytotoxicity potential for wheat in a natural acidic contaminated soil. Ecotoxicol. Environ. Saf..

[bib34] Oliveira B.R.M., de Almeida A.-A.F., Santos N. de A., Pirovani C.P. (2022). Tolerance strategies and factors that influence the cadmium uptake by cacao tree. Sci. Hortic. (Amsterdam).

[bib35] O’Connor D., Peng T., Zhang J., Tsang D.C.W., Alessi D.S., Shen Z., Bolan N.S., Hou D. (2018). Biochar application for the remediation of heavy metal polluted land: a review of *in situ* field trials. Sci. Total Environ..

[bib36] Pan J., Plant J.A., Voulvoulis N., Oates C.J., Ihlenfeld C. (2009). Cadmium levels in Europe: implication for human health. Environ. Geochem. Health.

[bib37] Pan Y., Koopmans G.F., Bonten L.T.C., Song J., Luo Y., Temminghoff E.J.M., Comans R.N.J. (2016). Temporal variability in trace metal solubility in a paddy soil not reflected in uptake by rice (*Oryza sativa* L.). Environ. Geochem. Health.

[bib38] Qian T.T., Wu P., Qin Q.Y., Huang Y.N., Wang Y.J., Zhou D.M. (2019). Screening of wheat straw biochars for the remediation of soils polluted with Zn (II) and Cd (II). J. Hazard Mater..

[bib39] Ramtahal G., Umaharan P., Hanuman A., Davis C., Ali L. (2019). The effectiveness of soil amendments, biochar and lime, in mitigating cadmium bioaccumulation in *Theobroma cacao* L. Sci. Total Environ..

[bib40] Rizwan M., Ali S., Abbas T., Zia-ur-Rehman M., Hannan F., Keller C., Al-Wabel M.I., Ok Y.S. (2016). Cadmium minimization in wheat: a critical review. Ecotoxicol. Environ. Saf..

[bib41] Shen X., Huang D.-Y., Ren X.-F., Zhu H.-H., Wang S., Xu C., He Y.-B., Luo Z.-C., Zhu Q.-H. (2016). Phytoavailability of Cd and Pb in crop straw biochar-amended soil is related to the heavy metal content of both biochar and soil. J. Environ. Manag..

[bib42] Shi R., Hong Z., Li J., Jiang J., Baquy M.A.-A., Xu R., Qian W. (2017). Mechanisms for increasing the pH buffering capacity of an acidic ultisol by crop residue-derived biochars. J. Agric. Food Chem..

[bib43] Shi R.-Y., Ni N., Nkoh J.N., Dong Y., Zhao W.-R., Pan X.-Y., Li J.-Y., Xu R.-K., Qian W. (2020). Biochar retards Al toxicity to maize (Zea mays L.) during soil acidification: the effects and mechanisms. Sci. Total Environ..

[bib44] Smolders E., Mertens J., Alloway B.J. (2013). Heavy Metals in Soils.

[bib45] Suhani I., Sahab S., Srivastava V., Singh R.P. (2021). Impact of cadmium pollution on food safety and human health. Curr. Opin. Toxicol..

[bib46] Uchimiya M., Klasson K.T., Wartelle L.H., Lima I.M. (2011). Influence of soil properties on heavy metal sequestration by biochar amendment: 1. Copper sorption isotherms and the release of cations. Chemosphere.

[bib47] Ullah I., Wang Y., Eide J.D., Dunwell M.J. (2018). Evolution, and functional analysis of natural resistance-associated macrophage proteins (NRAMPs) from Theobroma cacao and their role in cadmium accumulation. Sci. Rep..

[bib48] Vanderschueren R., Argüello D., Blommaert H., Montalvo D., Barraza F., Maurice L., Schreck E., Schulin R., Lewis C., Vazquez J.L., Umaharan P., Chavez E., Sarret G., Smolders E. (2021). Mitigating the level of cadmium in cacao products: reviewing the transfer of cadmium from soil to chocolate bar. Sci. Total Environ..

[bib49] Vanderschueren R., Doevenspeck J., Helsen F., Mounicou S., Santner J., Delcour A.J., Chavez E., Smolders E. (2022). Cadmium migration from nib to testa during cacao fermentation is driven by nib acidification. LWT.

[bib50] Wang Y.-H., Siu W.-K. (2006). Structure characteristics and mechanical properties of kaolinite soils. I. Surface charges and structural characterizations. Can. Geotech. J..

[bib51] Wang R.-Z., Huang D.-L., Liu Y.-G., Zhang C., Lai C., Zeng G.-M., Cheng M., Gong X.-M., Wan J., Luo H. (2018). Investigating the adsorption behavior and the relative distribution of Cd2+ sorption mechanisms on biochars by different feedstock. Bioresour. Technol..

[bib52] WHO (2011). Prepared by the Seventy-third Meeting of the Joint FAO/WHO Expert Committee on Food Additives (JECFA).

[bib53] Xiao R., Wang P., Mi S., Ali A., Liu X., Li Y., Guan W., Li R., Zhang Z. (2019). Effects of crop straw and its derived biochar on the mobility and bioavailability in Cd and Zn in two smelter-contaminated alkaline soils. Ecotoxicol. Environ. Saf..

[bib54] Yuan J.-H., Xu R.-K., Zhang H. (2011). The forms of alkalis in the biochar produced from crop residues at different temperatures. Bioresour. Technol..

[bib55] Yuan P., Wang J., Pan Y., Shen B., Wu C. (2019). Review of biochar for the management of contaminated soil: preparation, application and prospect. Sci. Total Environ..

[bib56] Zhang Y., Chen T., Liao Y., Reid B.J., Chi H., Hou Y., Cai C. (2016). Modest amendment of sewage sludge biochar to reduce the accumulation of cadmium into rice (*Oryza sativa* L.): a field study. Environ. Pollut..

