# Supplementary material for: Reducing cadmium bioaccumulation in Theobroma cacao using biochar: basis for scaling-up to field
Source: Heliyon. 2022 Jun 23;8(6):e09790. doi: 10.1016/j.heliyon.2022.e09790 (PMC9241039; doi:10.1016/j.heliyon.2022.e09790)
Supplement: Supplementary information [file mmc1.docx]

**Supplementary information for:**

**Reducing cadmium bioaccumulation in *Theobroma cacao* using biochar: basis for scaling-up to field**

Julián E. López^1^; Catalina Arroyave^1^; Adriana Aristizábal^2^; Byrone Almeida^3^, Santiago Builes^2^; Eduardo Chávez^3,4*^

^1^ Environmental Engineering Department, Universidad de Medellín, Carrera 87 # 30-65, 050026, Medellín, Colombia.

^2^ Process Engineering Department, Universidad EAFIT, Carrera 49 # 7 Sur-50, 050022 Medellín, Colombia.

^3^Universidad Estatal de Milagro, UNEMI, Facultad de Ingenierías, Ciudadela Universitaria km 1 ½, Milagro, Ecuador.

^4^ Escuela Superior Politécnica del Litoral, ESPOL, Facultad de Ciencias de la Vida, Campus Gustavo Galindo Km. 30.5 Vía Perimetral, P.O. Box 09-01-5863, Guayaquil, Ecuador.

*Corresponding author:

*E-mail address:* [fchavez@espol.edu.ec](mailto:fchavez@espol.edu.ec)


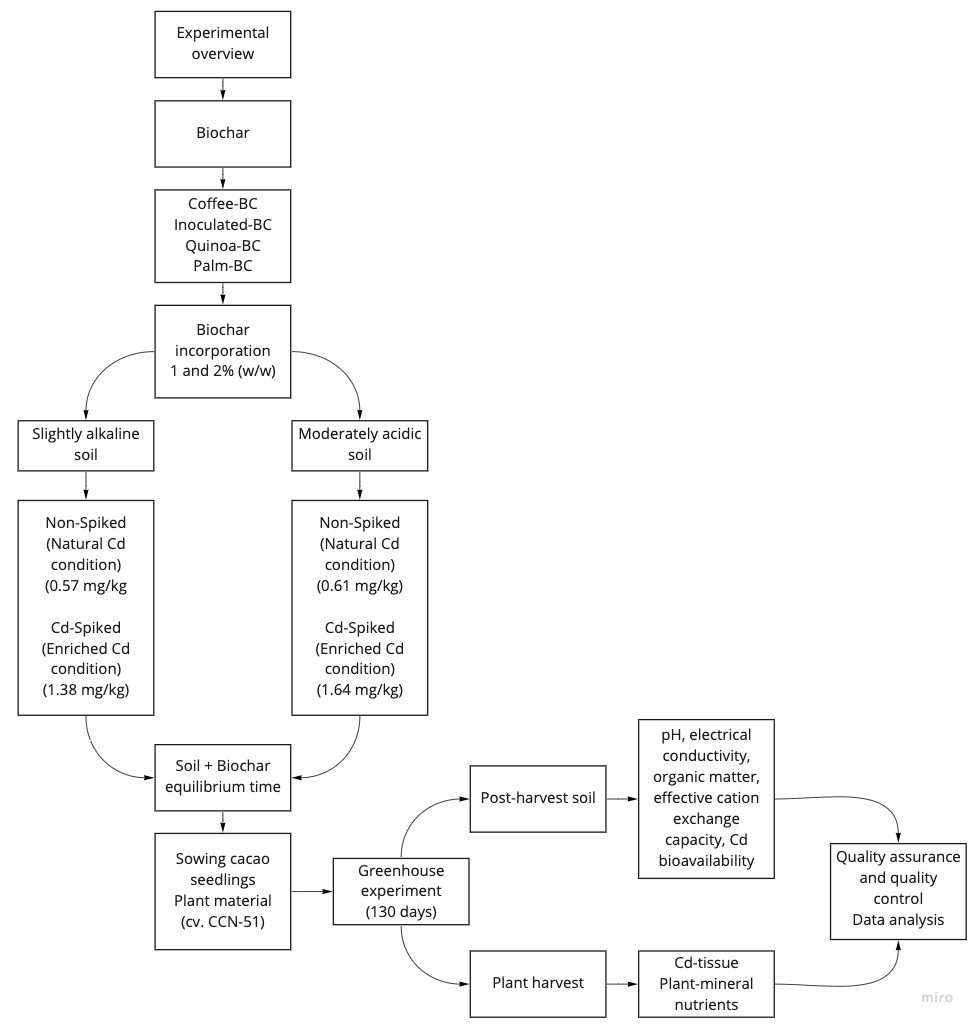


Fig. S1. Flowchart summarizing the biochar and soil preparation and the greenhouse experiments and parameters evaluated.

Supplementary Description S1

To assure the reliability of the results quality assurance and quality control protocols were included throughout the analytical processes. Certified reference materials (CRM) for soil (NIST 2709a) and plant (NIST 1573a) were included in pseudo-total soil and plant Cd analyses, respectively. In addition, internal reference materials, blanks and duplicates were analyzed every 20 samples. Ultrapure water was used in all the analysis. All reagents were of analytical grade, the glassware was soaked in an acid bath (3% v/v HNO_3_) overnight prior use. Calibration curve of ICP-OES was accepted at R^2^ > 0.9996 (for Cd). The average (± SD) Cd recoveries of the CRM were 103% (± 1.60) and 97% (± 2.00) for soil and plant materials, respectively. Limit of detection for Cd was 8 µg L^-1^.

Fig. S2. Soil pH dynamics during 130 days for slightly alkaline soils non-spiked with 1% (a) and 2 % (b) biochar, and Cd-spiked slightly alkaline soils with 1% (c) and 2 % (d) biochar and for moderately acidic soils non-spiked with 1 (e) and 2 % (f) biochar, and Cd-spiked moderately acidic soils with 1 (g) and 2 % (h) biochar. Error bars are standard error of the means (n=3). Asterisks above the bar indicate a significant (P<0.05) difference between the treatment and the control by Dunnett´s test.

Soil pH dynamics were evaluated at 15, 30, 60, 90 and 130 days as shown in Fig. S1. At the highest dose (2%), all biochars increased soil pH within the first 15 days, followed by a sharp decrease at 30 days and stabilization afterwards. In the first 15 days, Quinoa-BC treatment increased soil pH by 1.0 unit in slightly alkaline soils and by 1.5 units in moderately acidic soils. This is in agreement with previous reports, which found that soil pH increased rapidly after biochar application followed by a slight decrease and stabilization period (Prapagdee et al., 2014; Qi et al., 2018).

Table S1. FT-IR analysis for Coffee-BC, Inoculated-BC, Quinoa-BC, Palm-BC.

| Peak (cm^-1^) | Biochar | Functional group |  |
| --- | --- | --- | --- |
| 421 | Inoculated-BC, Palm-BC | Metal-oxygen bonds in octahedral complex | (Jung et al., 2017) |
| 870 | Coffee-BC, Quinoa-BC | CO_3_ | (Bashir et al., 2018) |
| 1030 | Quinoa-BC | Combination of aliphatic C-O peak and Si-O-Si peak | (Shen et al., 2019) |
| 1372 | Coffee-BC | C-N stretching | (Hestrin et al., 2019) |
| 1379 | Quinoa-BC | C-H or CH_3_ bending | (Xiao et al., 2018) |
| 1600  2900 | Quinoa-BC | Stretching modes of aromatics with multiple OH substitutions | (Gao et al., 2019) |
| 3182 | Quinoa-BC | N-H stretches | (Liu et al., 2018) |
| 3043 | Coffee-BC | C-H stretching | (Luo et al., 2017) |

Table S1 shows the result of the FTIR analysis for the four biochar. Quinoa-BC had functional groups previously related to the immobilization of cadmium in soils. The peak at 870 cm^−1^ corresponds to contents of CaCO_3_, which might cause significant immobilization of cadmium due to precipitation as CdCO_3_ and the presence of peak 1030 cm^-1^ (Si-O) can contribute to stabilize cadmium (Bashir et al., 2018). Additionally, the functional groups hydroxyl, carboxylate and phenolic hydroxyl, play an important role in Cd adsorption via Cd-ligands coordination bonds (Gao et al., 2019).

Table S2. Independent and combined effects of biochar type and application dose on soil physicochemical properties. Significant levels: * (*P<0.05*); ** (*P<0.01*).

|  | pH | CECe (cmol_c_ kg^-1^) | EC (dS m^-1^) | SOM (%) |
| --- | --- | --- | --- | --- |
| Non-Spiked  slightly alkaline soil |  |  |  |  |
|  |  |  |  |  |
| Biochar | 0.0707 | 0.0015** | <0.001** | 0.0002** |
| Dose | 0.0016** | 0.4266 | <0.001** | <0.001** |
| Biochar x Dose | 0.9366 | 0.0285* | <0.001** | 0.8395 |
|  |  |  |  |  |
| Cd-Spiked  slightly alkaline soil |  |  |  |  |
|  |  |  |  |  |
| Biochar | 0.3334 | 0.9190 | <0.0001** | 0.0175* |
| Dose | 0.5245 | 0.8115 | 0.0052** | <0.0001** |
| Biochar x Dose | 0.7556 | 0.9524 | 0.0051** | 0.0935 |
|  |  |  |  |  |
| Non-Spiked  moderately acidic soil |  |  |  |  |
|  |  |  |  |  |
| Biochar | 0.0001** | <0.0001** | <0.001** | 0.0071** |
| Dose | 0.0372* | 0.0026** | <0.001** | <0.0001** |
| Biochar x Dose | 0.1414 | 0.0087** | <0.001** | 0.1002 |
|  |  |  |  |  |
| Cd-Spiked  moderately acidic soil |  |  |  |  |
|  |  |  |  |  |
| Biochar | <0.0001** | 0.0048** | <0.001** | 0.0100** |
| Dose | 0.0722 | 0.6664 | <0.001** | <0.0001** |
| Biochar x Dose | 0.0138* | 0.0093** | <0.001** | 0.4461 |

SOM: soil organic matter. CECe: effective cation exchange capacity. EC: electrical conductivity.

Table S3. Independent and combined effects of biochar type and application dose on concentration and reduction factor (Rf) in Cd pools. Significant levels: * (*P<0.05*); ** (*P<0.01*).

|  | CaCl_2_-Cd (mg kg^-1^) | NH_4_AOc-Cd (mg kg^-1^) | HCl-Cd (mg kg^-1^) | Rf  CaCl_2_-Cd | Rf  NH_4_AOc-Cd | Rf  HCl-Cd |
| --- | --- | --- | --- | --- | --- | --- |
| Non-Spiked  slightly alkaline soil |  |  |  |  |  |  |
|  |  |  |  |  |  |  |
| Biochar | - | <0.0001** | 0.4886 | - | <0.0001** | 0.4648 |
| Dose | - | 0.0031** | 0.0579 | - | 0.0001** | 0.0567 |
| Biochar x Dose | - | 0.0133* | 0.6111 | - | 0.0037* | 0.5483 |
|  |  |  |  |  |  |  |
| Cd-Spiked  slightly alkaline soil |  |  |  |  |  |  |
|  |  |  |  |  |  |  |
| Biochar | <0.0001** | 0.0018** | 0.0009** | <0.0001** | 0.0004** | 0.0009** |
| Dose | 0.0056** | 0.6311 | 0.9245 | <0.0001** | 0.2872 | 0.9696 |
| Biochar x Dose | 0.0405* | 0.0051** | 0.8086 | <0.0001** | 0.0026** | 0.8406 |
|  |  |  |  |  |  |  |
| Non-Spiked  moderately acidic soil |  |  |  |  |  |  |
|  |  |  |  |  |  |  |
| Biochar | 0.0059** | <0.0001** | 0.7127 | 0.0004** | 0.0001** | 0.7213 |
| Dose | 0.0848 | <0.0001** | 0.2114 | 0.0067** | 0.0001** | 0.2000 |
| Biochar x Dose | 0.5676 | 0.1437 | 0.0622 | 0.0201* | 0.0320 | 0.0727 |
|  |  |  |  |  |  |  |
| Cd-Spiked  moderately acidic soil |  |  |  |  |  |  |
|  |  |  |  |  |  |  |
| Biochar | <0.0001** | 0.0001** | <0.0001** | <0.0001** | <0.0001** | <0.0001** |
| Dose | 0.0002** | 0.0691 | 0.2766 | <0.0001** | 0.0659 | 0.1872 |
| Biochar x Dose | 0.0145* | 0.2379 | 0.1873 | 0.0001** | 0.0568 | 0.3139 |

Table S4. Cd concentration in stems and roots at 130 days. Values are averages of three replicates. Different letters indicate statistical significance at *P* < 0.05 (*) and *P* < 0.01 (**) compared to the control.

| Soil | Treatment |  | Stem-Cd | Root-Cd |
| --- | --- | --- | --- | --- |
| Slightly alkaline | Non-spiked | Coffee-BC1% | 3.7 a | 1.0 a |
|  |  | Coffee-BC2% | 2.9 a | 1.1 a |
|  |  | Inoculated-BC1% | 3.4 a | 1.2 a |
|  |  | Inoculated-BC2% | 3.1 a | 1.4 a |
|  |  | Quinoa-BC1% | 2.8 a | 1.2 a |
|  |  | Quinoa-BC2% | 2.0 b* | 1.0 a |
|  |  | Palm-BC1% | 3.2 a | 1.2 a |
|  |  | Palm-BC2% | 2.6 a | 1.2 a |
|  |  | Control | 3.6 a | 1.3 a |
| Slightly alkaline | Cd-spiked | Coffee-BC1% | 5.7 a | 2.4 a |
|  |  | Coffee-BC2% | 5.0 a | 1.8 a |
|  |  | Inoculated-BC1% | 6.2 a | 2.1 a |
|  |  | Inoculated-BC2% | 3.8 a | 2.0 a |
|  |  | Quinoa-BC1% | 4.5 a | 1.8 a |
|  |  | Quinoa-BC2% | 3.7 a | 2.0 a |
|  |  | Palm-BC1% | 5.1 a | 2.1 a |
|  |  | Palm-BC2% | 4.1 a | 2.3 a |
|  |  | Control | 5.1 a | 2.3 a |
| Moderately acidic | Non-spiked | Coffee-BC1% | 5.0 a | 1.8 a |
|  |  | Coffee-BC2% | 4.3 a | 1.3 a |
|  |  | Inoculated-BC1% | 4.5 a | 1.7 a |
|  |  | Inoculated-BC2% | 5.2 b | 1.6 a |
|  |  | Quinoa-BC1% | 4.3 a | 1.7 a |
|  |  | Quinoa-BC2% | 3.2 b** | 1.5 a |
|  |  | Palm-BC1% | 4.9 a | 1.6 a |
|  |  | Palm-BC2% | 5.0 a | 2.0 a |
|  |  | Control | 4.1 a | 1.7 a |
| Moderately acidic | Cd-spiked | Coffee-BC1% | 12.7 a | 4.6 a |
|  |  | Coffee-BC2% | 7.7 b* | 3.2 a |
|  |  | Inoculated-BC1% | 17.6 a | 6.8 a |
|  |  | Inoculated-BC2% | 14.5 a | 4.1 a |
|  |  | Quinoa-BC1% | 7.8 b* | 5.0 a |
|  |  | Quinoa-BC2% | 8.0 b* | 3.3 a |
|  |  | Palm-BC1% | 14.2 a | 3.3 a |
|  |  | Palm-BC2% | 11.6 a | 3.7 a |
|  |  | Control | 13.7 a | 5.0 a |

Table S5. Independent and combined effects of biochar type and application dose on roots-, stem- and leaf-Cd and reduction factor (Rf) in leaf-Cd. Significant levels: * (*P<0.05*); ** (*P<0.01*).

|  | Roots-Cd  (mg kg^-1^) | Stem-Cd  (mg kg^-1^) | Leaf-Cd  (mg kg^-1^) | Rf leaf-Cd |
| --- | --- | --- | --- | --- |
| Non-spiked  slightly alkaline soil |  |  |  |  |
|  |  |  |  |  |
| Biochar | 0.1779 | 0.1522 | 0.2195 | 0.0901 |
| Dose | 0.7625 | 0.0516 | 0.2610 | 0.1585 |
| Biochar x Dose | 0.2666 | 0.9492 | 0.2188 | 0.0901 |
|  |  |  |  |  |
| Cd-Spiked  slightly alkaline soil |  |  |  |  |
|  |  |  |  |  |
| Biochar | 0.5464 | 0.0580 | 0.1039 | 0.1219 |
| Dose | 0.6536 | 0.0007** | 0.0013** | 0.0009** |
| Biochar x Dose | 0.3654 | 0.1202 | 0.3495 | 0.2974 |
|  |  |  |  |  |
| Non-spiked  moderately acidic soil |  |  |  |  |
|  |  |  |  |  |
| Biochar | 0.6560 | 0.0008** | 0.0008** | <0.0001** |
| Dose | 0.3675 | 0.2437 | 0.2371 | 0.0245* |
| Biochar x Dose | 1.9165 | 0.0081** | 0.3234 | 0.0474* |
|  |  |  |  |  |
| Cd-Spiked  moderately acidic soil |  |  |  |  |
|  |  |  |  |  |
| Biochar | 0.0032** | <0.0001** | 0.0008** | 0.0089** |
| Dose | 0.0049** | 0.0175* | 0.0194* | 0.0611 |
| Biochar x Dose | 0.0033** | 0.0186* | 0.0840 | 0.0478* |

Fig. S3. Leaf-Cd as function of (a) stem-Cd, and (b) root-Cd. Stem-Cd as function of (c) root-Cd. r represents the correlation coefficient. The data correspond to the leaf, stem, and root Cd concentration for soils with slightly alkaline soil and moderately acidic soil (n=108).

Table S6. Leaf-Cd reduction factor (Rf) values at 130 days. Values are average of three replicates (±SE). Different letters indicate statistical significance at *P* < 0.05 (Tukey´s test).

| Soil | Treatment | | Rf  Leaf-Cd |
| --- | --- | --- | --- |
| Slightly alkaline | Non-spiked | Coffee-BC1% | 1.0 ± 0.1 b |
|  |  | Coffee-BC2% | 1.2 ± 0.1 ab |
|  |  | Inoculated-BC1% | 1.1 ± 0.1 ab |
|  |  | Inoculated-BC2% | 1.0 ± 0.2 ab |
|  |  | Quinoa-BC1% | 1.1 ± 0.1 ab |
|  |  | Quinoa-BC2% | 1.7 ± 0.3 a |
|  |  | Palm-BC1% | 1.3 ± 0.2 ab |
|  |  | Palm-BC2% | 1.2 ± 0.1 ab |
| Slightly alkaline | Spiked | Coffee-BC1% | 0.9 ± 0.1 ab |
|  |  | Coffee-BC2% | 1.0 ± 0.1 ab |
|  |  | Inoculated-BC1% | 0.9 ± 0.1 ab |
|  |  | Inoculated-BC2% | 1.1 ± 0.1 ab |
|  |  | Quinoa-BC1% | 1.0 ± 0.1 ab |
|  |  | Quinoa-BC2% | 1.2 ± 0.0 a |
|  |  | Palm-BC1% | 0.8 ± 0.1 b |
|  |  | Palm-BC2% | 1.1 ± 0.1 ab |
| Moderately acidic | Non-spiked | Coffee-BC1% | 1.1 ± 0.1 b |
|  |  | Coffee-BC2% | 1.3 ± 0.1 b |
|  |  | Inoculated-BC1% | 1.0 ± 0.1 b |
|  |  | Inoculated-BC2% | 1.0 ± 0.1 b |
|  |  | Quinoa-BC1% | 1.3 ± 0.1 b |
|  |  | Quinoa-BC2% | 1.8 ± 0.1 a |
|  |  | Palm-BC1% | 0.9 ± 0.0 b |
|  |  | Palm-BC2% | 0.9 ± 0.1 b |
| Moderately acidic | Spiked | Coffee-BC1% | 1.0 ± 0.0 cd |
|  |  | Coffee-BC2% | 1.5 ± 0.1 bc |
|  |  | Inoculated-BC1% | 0.8 ± 0.1 d |
|  |  | Inoculated-BC2% | 1.0 ± 0.1 cd |
|  |  | Quinoa-BC1% | 1.1 ± 0,.1 cd |
|  |  | Quinoa-BC2% | 2.2 ± 0.2 a |
|  |  | Palm-BC1% | 1.4 ± 0.1 bcd |
|  |  | Palm-BC2% | 1.8 ± 0.3 ab |

a

b

c


Fig. S4. Comparation of Rf values of the studied biochar and selected literature (Houben et al., 2013; Mohamed et al., 2018; Xiao et al., 2019) calculated Rf as function of the ash (%) (a), alkaline elements (meq kg^-1^) (b), and EC (dS m^-1^) (c). Circle: literature data. Triangle: Coffee-BC. Square: Inoculated-BC. Rectangle: Quinoa-BC. Diamond: Palm-BC. The Rf values for studied biochar were calculated as the average of the Rfs for all treatments of the respective biochar.

Table S7. Correlation coefficients of biochar properties and Cd bioavailability (CaCl_2_-, NH_4_OAc-, and HCl-Cd). Statistical significance is represented by **P* < 0.05, ***P* < 0.01.

|  | Ash  Biochar | Ca  Biochar | Mg  Biochar | K  Biochar | Na  Biochar | EC  Biochar | pH  Biochar | C  Biochar | CaCl_2_-Cd | HCl-Cd | NH_4_OAc-Cd |
| --- | --- | --- | --- | --- | --- | --- | --- | --- | --- | --- | --- |
| Ash Biochar |  | 0.77** | 0.77** | 0.66** | 0.74** | 0.71** | 0.82** | -0.92** | -0.68** | -0.81** | -0.84** |
| Ca Biochar |  |  | 0.91** | 0.94** | 0.86** | 0.98** | 0.99** | -0.94** | -0.29 | -0.85** | -0.58** |
| Mg Biochar |  |  |  | 0.70** | 0.99** | 0.79** | 0.91** | -0.95** | -0.30 | -0.96** | -0.58** |
| K Biochar |  |  |  |  | 0.62** | 0.99** | 0.93** | -0.81** | -0.24 | -0.63** | -0.50* |
| Na Biochar |  |  |  |  |  | 0.72** | 0.86** | -0.92** | -0.29 | -0.96** | -0.56** |
| EC Biochar |  |  |  |  |  |  | 0.51** | -0.87** | -0.24 | -0.36 | -0.32 |
| pH Biochar |  |  |  |  |  |  |  | -0.96** | -0.27 | 0.22 | -0.09 |
| C Biochar |  |  |  |  |  |  |  |  | 0.47 | 0.93** | 0.73** |
| CaCl_2_ -Cd |  |  |  |  |  |  |  |  |  | 0.51** | 0.84** |
| HCl-Cd |  |  |  |  |  |  |  |  |  |  | 0.78** |
| NH_4_OAc-Cd |  |  |  |  |  |  |  |  |  |  |  |

EC: Electrical conductivity.

Table S8. Growth parameters and plant-P and plant-K concentration in cacao plants at 130 days. Values are the means of three replicates. Different letters indicate statistical significance at *P* < 0.05 (*) and *P* < 0.01 (**) compared to the control (Dunnett´s test).

| Soil | Treatment | |  | Plant dry biomass  (g) | Stem diameter  (mm) | Stem length  (cm) | Root length  (cm) | Plant-P  (g kg^-1^) | Plant-K  (g kg^-1^) |
| --- | --- | --- | --- | --- | --- | --- | --- | --- | --- |
| Slightly alkaline | Non-spiked | Coffee-BC1% |  | 23.4 a | 7.0 a | 55.3 a | 43.0 a | 1.84 a | 14.9 a |
|  |  | Coffee-BC2% |  | 22.2 a | 8.0 a | 49.3 a | 27.3 a | 1.67 a | 14.4 a |
|  |  | Inoculated-BC1% |  | 22.0 a | 7.3 a | 53.9 a | 32.7 a | 1.77 a | 13.5 a |
|  |  | Inoculated-BC% |  | 28.1 a | 9.0 a | 58.9 a | 39.7 a | 1.68 a | 15.2 a |
|  |  | Quinoa-BC1% |  | 23.3 a | 9.0 a | 58.5 a | 32.7 a | 1.95 a | 15.8 a |
|  |  | Quinoa-BC2% |  | 20.4 a | 9.0 a | 58.2 a | 37.3 a | 1.81 a | 18.1 b* |
|  |  | Palm-BC1% |  | 24.7 a | 7.3 a | 53.0 a | 32.7 a | 1.80 a | 15.3 a |
|  |  | Palm-BC2% |  | 25.0 a | 8.0 a | 54.7 a | 28.0 a | 2.02 a | 15.6 a |
|  |  | Control |  | 26.0 a | 8.3 a | 53.7 a | 30.7 a | 2.00 a | 15.0 a |
| Slightly alkaline | Cd-spiked | Coffee-BC1% |  | 23.6 a | 9.0 a | 51.0 a | 31.7 a | 2.11 a | 16.9 a |
|  |  | Coffee-BC2% |  | 22.5 a | 8.6 a | 57.2 a | 31.0 a | 1.91 a | 14.9 a |
|  |  | Inoculated-BC1% |  | 25.9 a | 7.6 a | 51.8 a | 30.7 a | 1.89 a | 15.5 a |
|  |  | Inoculated-BC% |  | 24.8 a | 8.3 a | 56.5 a | 29.7 a | 1.95 a | 16.8 a |
|  |  | Quinoa-BC1% |  | 25.7 a | 8.3 a | 55.2 a | 39.7 a | 1.97 a | 16.4 a |
|  |  | Quinoa-BC2% |  | 24.2 a | 8.0 a | 60.3 a | 29.0 a | 2.08 a | 17.8 b* |
|  |  | Palm-BC1% |  | 22.2 a | 8.6 a | 56.5 a | 27.7 a | 1.79 a | 15.1 a |
|  |  | Palm-BC2% |  | 21.6 a | 8.0 a | 56.2 a | 32.0 a | 1.80 a | 14.2 a |
|  |  | Control |  | 26.9 a | 8.3 a | 50.8 a | 29.7 a | 1.81 a | 13.2 a |
| Moderately acidic | Non-spiked | Coffee-BC1% |  | 26.6 a | 8.6 a | 55.8 a | 29.0 a | 1.01 a | 11.5 a |
|  |  | Coffee-BC2% |  | 26.6 a | 8.0 a | 50.0 a | 29.3 a | 1.23 a | 17.1 b* |
|  |  | Inoculated-BC1% |  | 24.6 a | 9.0 a | 59.0 a | 27.3 a | 1.00 a | 10.0 a |
|  |  | Inoculated-BC% |  | 25.5 a | 8.0 a | 52.5 a | 24.0 a | 1.11 a | 11.9 a |
|  |  | Quinoa-BC1% |  | 22.2 a | 9.0 a | 50.0 a | 30.0 a | 1.22 a | 17.0 b* |
|  |  | Quinoa-BC2% |  | 25.0 a | 8.0 a | 50.7 a | 26.3 a | 1.52 b* | 20.8 b** |
|  |  | Palm-BC1% |  | 26.3 a | 9.0 a | 55.5 a | 22.0 a | 1.03 a | 11.1 a |
|  |  | Palm-BC2% |  | 28.1 a | 8.3 a | 53.0 a | 25.3 a | 1.22 a | 12.8 a |
|  |  | Control |  | 27.3 a | 8.6 a | 56.0 a | 32.3 a | 1.03 a | 12.5 a |
| Moderately acidic | Cd-spiked | Coffee-BC1% |  | 25.1 a | 8.6 a | 58.0 a | 32.7 a | 1.15 a | 17.0 b* |
|  |  | Coffee-BC2% |  | 25.3 a | 8.0 a | 53.0 a | 26.0 a | 1.56 b* | 19.1 b** |
|  |  | Inoculated-BC1% |  | 30.0 a | 9.3 a | 65.2 a | 30.0 a | 1.20 a | 14.1 a |
|  |  | Inoculated-BC% |  | 25.9 a | 8.3 a | 56.0 a | 24.7 a | 1.03 a | 13.8 a |
|  |  | Quinoa-BC1% |  | 22.9 a | 8.0 a | 55.2 a | 25.7 a | 1.26 a | 13.8 a |
|  |  | Quinoa-BC2% |  | 25.1 a | 8.3 a | 51.0 a | 30.7 a | 1.57 b* | 16.6 b* |
|  |  | Palm-BC1% |  | 27.9 a | 10.0 a | 57.7 a | 26.0 a | 1.26 a | 14.9 a |
|  |  | Palm-BC2% |  | 26.9 a | 7.6 a | 56.5 a | 24.7 a | 1.53 b* | 16.6 b* |
|  |  | Control |  | 27.2 a | 8.6 a | 56.7 a | 23.3 a | 1.08 a | 12.6 a |

**References**

Bashir, S., Hussain, Q., Shaaban, M., Hu, H., 2018. Efficiency and surface characterization of different plant derived biochar for cadmium (Cd) mobility, bioaccessibility and bioavailability to Chinese cabbage in highly contaminated soil. Chemosphere 211, 632–639. https://doi.org/10.1016/j.chemosphere.2018.07.168

Gao, X., Peng, Y., Zhou, Y., Adeel, M., Chen, Q., 2019. Effects of magnesium ferrite biochar on the cadmium passivation in acidic soil and bioavailability for packoi (Brassica chinensis L.). J. Environ. Manage. 251, 109610. https://doi.org/10.1016/j.jenvman.2019.109610

Hestrin, R., Torres-Rojas, D., Dynes, J.J., Hook, J.M., Regier, T.Z., Gillespie, A.W., Smernik, R.J., Lehmann, J., 2019. Fire-derived organic matter retains ammonia through covalent bond formation. Nat. Commun. 10, 664. https://doi.org/10.1038/s41467-019-08401-z

Houben, D., Evrard, L., Sonnet, P., 2013b. Mobility, bioavailability and pH-dependent leaching of cadmium, zinc and lead in a contaminated soil amended with biochar. Chemosphere 92, 1450–1457. https://doi.org/10.1016/j.chemosphere.2013.03.055

Jung, K.-W., Lee, S., Lee, Y.J., 2017. Synthesis of novel magnesium ferrite (MgFe2O4)/biochar magnetic composites and its adsorption behavior for phosphate in aqueous solutions. Bioresour. Technol. 245, 751–759. https://doi.org/10.1016/j.biortech.2017.09.035

Liu, D., Lu, C., Wu, J., 2018. CuO/g-C3N4 nanocomposite for elemental mercury capture at low temperature. J. Nanoparticle Res. 20, 277. https://doi.org/10.1007/s11051-018-4374-4

Luo, X., Yu, H., Xi, Y., Fang, L., Liu, L., Luo, J., 2017. Selective removal Pb( <scp>ii</scp> ) ions form wastewater using Pb( <scp>ii</scp> ) ion-imprinted polymers with bi-component polymer brushes. RSC Adv. 7, 25811–25820. https://doi.org/10.1039/C7RA03536E

Mohamed, I., Ali, M., Ahmed, N., Abbas, M.H.H., Abdelsalam, M., Azab, A., Raleve, D., Fang, C., 2018. Cow manure-loaded biochar changes Cd fractionation and phytotoxicity potential for wheat in a natural acidic contaminated soil. Ecotoxicol. Environ. Saf. 162, 348–353. https://doi.org/10.1016/j.ecoenv.2018.06.065

Prapagdee, S., Piyatiratitivorakul, S., Petsom, A., Tawinteung, N., 2014. Application of Biochar for Enhancing Cadmium and Zinc Phytostabilization in Vigna radiata L. Cultivation. Water, Air, Soil Pollut. 225, 2233. https://doi.org/10.1007/s11270-014-2233-1

Qi, F., Lamb, D., Naidu, R., Bolan, N.S., Yan, Y., Ok, Y.S., Rahman, M.M., Choppala, G., 2018. Cadmium solubility and bioavailability in soils amended with acidic and neutral biochar. Sci. Total Environ. 610–611, 1457–1466. https://doi.org/10.1016/j.scitotenv.2017.08.228

Shen, Z., Fan, X., Hou, D., Jin, F., O’Connor, D., Tsang, D.C.W., Ok, Y.S., Alessi, D.S., 2019. Risk evaluation of biochars produced from Cd-contaminated rice straw and optimization of its production for Cd removal. Chemosphere 233, 149–156. https://doi.org/10.1016/j.chemosphere.2019.05.238

Xiao, B., Dai, Q., Yu, X., Yu, P., Zhai, S., Liu, R., Guo, X., Liu, J., Chen, H., 2018. Effects of sludge thermal-alkaline pretreatment on cationic red X-GRL adsorption onto pyrolysis biochar of sewage sludge. J. Hazard. Mater. 343, 347–355. https://doi.org/10.1016/j.jhazmat.2017.10.001

Xiao, R., Wang, P., Mi, S., Ali, A., Liu, X., Li, Y., Guan, W., Li, R., Zhang, Z., 2019. Effects of crop straw and its derived biochar on the mobility and bioavailability in Cd and Zn in two smelter-contaminated alkaline soils. Ecotoxicol. Environ. Saf. 181, 155–163. https://doi.org/10.1016/j.ecoenv.2019.06.005
